# Spatially Programmable Electroadhesive Enables In Situ Site‐Selective Functional Coupling

**DOI:** 10.1002/adma.202511039

**Published:** 2025-10-14

**Authors:** Yuting Guo, Zhuoming Liang, Guoshi Xu, Zhen Gu, Yuheng Xu, Michael Raphael Panganiban, Xue Cai, Jet Yong Kiat Lim, Jiguang Zhang, Jingxiu Huang, Tingting Fan, Qi Gu, Yuxin Liu

**Affiliations:** ^1^ Department of Biomedical Engineering National University of Singapore Singapore 117583 Singapore; ^2^ The N.1 Institute for Health National University of Singapore Singapore 117456 Singapore; ^3^ Institute for Health Innovation and Technology (iHealthtech) National University of Singapore Singapore 119276 Singapore; ^4^ Human Organ Physiopathology Emulation System State Key Laboratory of Organ Regeneration and Reconstruction Institute of Zoology Chinese Academy of Sciences Chaoyang District Beijing 100101 China; ^5^ Beijing Institute for Stem Cell and Regenerative Medicine Chaoyang District Beijing 100101 China; ^6^ University of Chinese Academy of Sciences Huairou District Beijing 100049 China; ^7^ School of Chemistry and Biological Engineering University of Science and Technology Beijing Beijing 100083 China; ^8^ Department of Chemical and Biomolecular Engineering National University of Singapore Singapore 117585 Singapore; ^9^ Department of Anesthesiology State Key Laboratory of Oncology in South China Guangdong Provincial Clinical Research Center for Cancer Sun Yat‐sen University Cancer Center Guangzhou 510060 China

**Keywords:** bioadhesive interfaces, mechanical and electrical coupling, spatially programmable

## Abstract

Precise intraoperative integration of bioelectronic devices with wet tissue surfaces remains a challenge due to the limited spatial control of adhesion sites. Here, an in situ spatially programmable electrical bioadhesive (termed “STICH”) is reported that enables site‐selective adhesion and functional coupling via light‐activated bonding with wet biological tissue. Upon irradiation with patterned green light, Rose Bengal in a chitosan/silver nanowire hydrogel matrix generates singlet oxygen, which oxidizes amino acid residues into carbonyl groups on the tissue surface. The covalent bonding is then formed between the newly formed reactive carbonyl group and amine groups on chitosan. The spatially programmable adhesive allows robust tissue bonding with a lap‐shear strength of 160 kPa and precise adhesion regions at ≈2 µm resolution. The light‐patternable adhesive enables spatially resolved mechanical coupling for directional electromechanical sensing on ex vivo cardiac tissue. The low impedance adhesive interface also provides spatially programmed electrical coupling for in vivo neuromuscular stimulation on intraoperatively selected muscle groups. This platform advances microscale device‐tissue integration and paves the way for reconfigurable bioelectronic therapies.

## Graphical abstract

A light‐activated, in situ spatially programmable bioadhesive (STICH) enables microscale, site‐selective integration of bioelectronic devices with wet tissue. It achieves robust mechanical bonding and low‐impedance electrical coupling, enabling reconfigurable neuromodulation and directional electromechanical sensing in vivo and ex vivo.

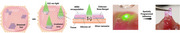

## 1 Introduction

Implantable bioelectronic devices have been widely explored for the treatment and monitoring of various conditions, ranging from cardiovascular diseases to neurological disorders.^[^ ^1^, ^2^, ^3^, ^4^ ^]^ Recent advances in integrating conductive and semiconductive functionalities into hydrogel‐based biointerfaces have broadened our capability to form functional coupling to biological tissue.^[^ ^5^, ^6^ ^]^ A stable tissue–device interface is essential for reliable signal acquisition and precise electrical or optical stimulation. Thin‐film electronic implants typically adhere to tissue via non‐covalent interactions such as van der Waals force^[^ ^7^ ^]^ or capillary forces.^[^ ^8^ ^]^ However, these weak bonds are easily disrupted by the cardiorespiratory cycle and organ motion, leading to unstable signal recordings and simulation. To improve interface stability, covalent bonding between tissue and nanocomposite hydrogels was used to enable strong adhesion and reliable bidirectional electrical communication.^[^ ^9^ ^]^ For example, NHS (N‐hydroxysuccinimide) ester groups in adhesive hydrogels were designed to react with primary amine groups on the tissue surface, forming a robust covalent bioadhesive interface.^[^ ^10^, ^11^, ^12^ ^]^

Despite the strong adhesion achieved using these covalent bonding strategies, spatiotemporal control of adhesion sites remains limited. For instance, EDC/NHS coupling chemistry initiates as soon as the adhesive contacts with tissue, leaving a short time window for repositioning the device during surgery. Moreover, achieving spatially selective bonding in situ remains a challenge. Pre‐fabricated adhesive cardiac patches for electrocoupling therapy are typically placed over a large area of the heart without distinguishing diseased regions from healthy tissue.^[^ ^13^ ^]^ In many conditions, such as myocardial infarction, cardiac arrhythmia, or epilepsy, the pathological regions do not precisely follow pre‐defined electrode patterns and can only be identified intraoperatively via electrophysiological mapping or imaging.^[^ ^14^, ^15^, ^16^ ^]^ Although implantable multi‐electrode arrays have multiple stimulation or recording sites, predetermined electrode locations cannot be repositioned intraoperatively to reach irregularly situated disease foci at high spatial resolution.^[^ ^17^, ^18^ ^]^

**Figure 1:**
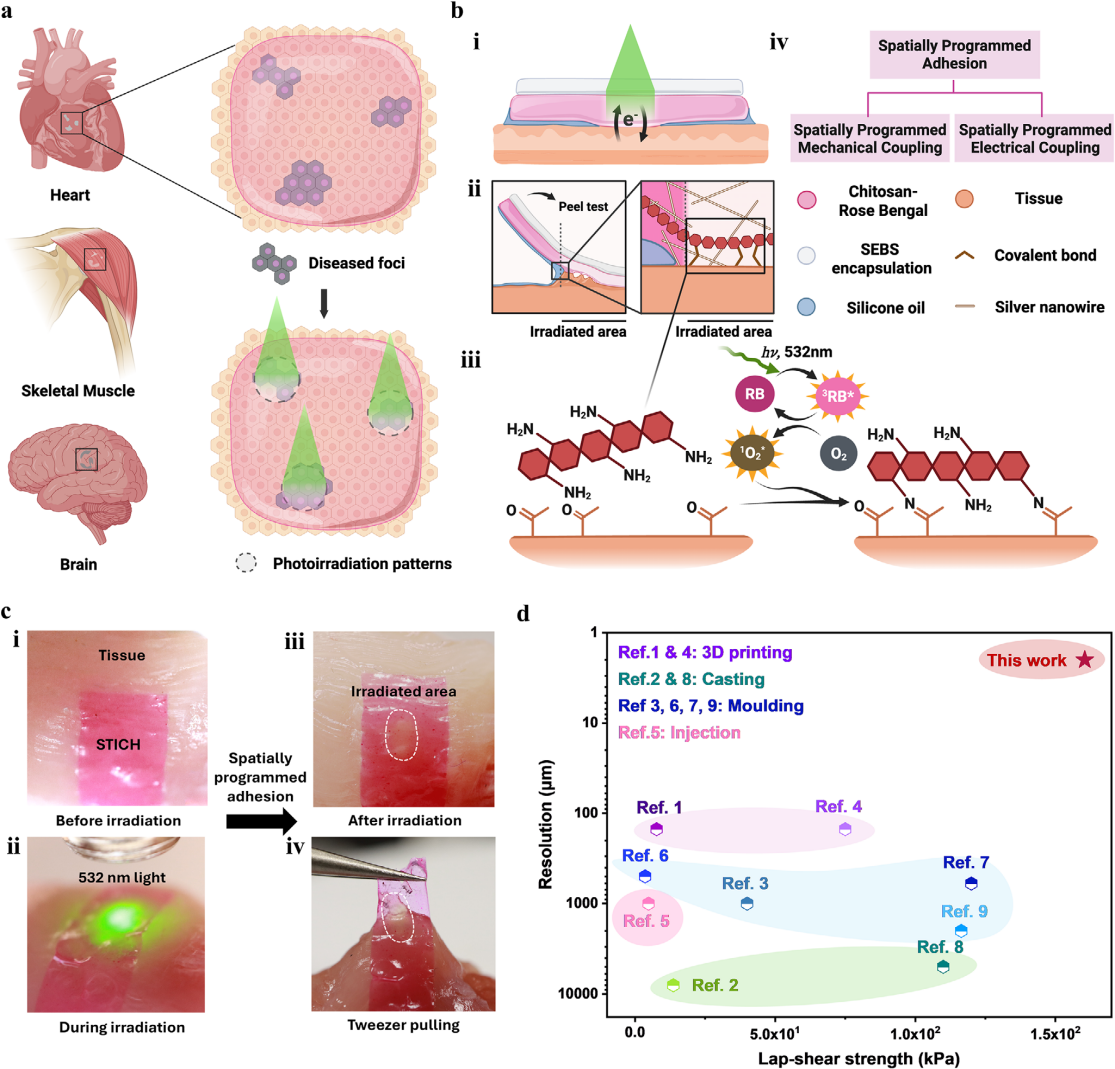
In situ spatially programmable electrical bioadhesive (STICH) interface. a) Schematic illustration of electrical bioadhesive interface for spatially programmable patterning. b) The design and adhesion mechanism of the STICH interface. i) The cross‐section of the spatially programmed bioadhesive interface; ii) Schematic illustration of the spatially programmed bioadhesive interface under peeling; iii) The formation of covalent bonds at the spatially programmed bioadhesive interface; iv) The applications of the spatially programmed adhesion. c) Spatially programmed adhesion of STICH on muscles. i) STICH interface before irradiation; ii) STICH interface during green light irradiation; iii) Irradiated area (marked by dashed circle) after irradiation; iv) STICH pulled by tweezers at spatially programmed bioadhesive interface. d) The comparison of lap‐shear strength and patterning resolution of bioadhesive interface reported in references and this work. (Ref.1,^[^ ^19^ ^]^ Ref.2,^[^ ^13^ ^]^ Ref.3,^[^ ^9^ ^]^ Ref.4,^[^ ^20^ ^]^ Ref.5,^[^ ^21^ ^]^ Ref.6,^[^ ^22^ ^]^ Ref.7,^[^ ^23^ ^]^ Ref.8,^[^ ^24^ ^]^ Ref.9,^[^ ^25^ ^]^).

To address these limitations, we propose a general strategy for functional coupling, including both electrical coupling and mechanical coupling, on wet biological tissue with high temporospatial control. The in situ spatially programmable electrical bioadhesive (termed “STICH”) interface relies on light‐controlled Rose Bengal‐photosensitized cross‐linking between the primary amine groups on the STICH and the oxidized carbonyl groups on biological tissues. Projected green‐light patterns, aligned according to the surgically identified diseased region, can be in situ converted directly into covalent adhesion domains with microscale precision, enabling robust, on‐demand, site‐selective tissue‐device coupling during the surgical procedure (**Figure** **1**a).

## 2 Results and Discussion

### 2.1 Design and Mechanism of STICH interface

Localized damage or pathological changes to organ surfaces, such as cardiac fibrosis following myocardial infarction,^[^ ^26^ ^]^ can disrupt the native cardiac conduction system. In such scenarios, the STICH interface can be designed to selectively reconstruct conductive pathways through spatially programmable patterning during surgery, offering a promising strategy for effective therapeutic intervention (Figure 1a). Compared to previously reported instant adhesive patches,^[^ ^27^, ^28^ ^]^ the green light‐triggered STICH interface offers advantages in both precise control over the adhesion pattern and an extended operational time window during surgery. In addition, adhesion is patterned exclusively within the illuminated regions and therefore forms spatially selective electrical contact with biological tissues.

To precisely interface with disease foci on various electrogenic organ surfaces, we designed the STICH interface to selectively form mechanical and electrical coupling through spatially programmable patterning during surgery (Figure 1a). We introduced a photosensitizer, Rose Bengal (RB), into a chitosan polymer matrix to allow light‐triggerable response^[^ ^29^, ^30^ ^]^ and spatial pattern ability. Upon exposure to green light at targeted locations, RB undergoes a photobleaching effect,^[^ ^31^, ^32^ ^]^ leading to visible decoloration in the irradiated regions (Figure S5, Supporting Information). When the STICH contacts the hydrated tissue surface, hydroxyl groups within the chitosan matrix absorb interfacial water and form physical interactions via hydrogen bonding and electrostatic forces. Thereafter, we use green light to excite locally diffused RB into the triplet state (^3^RB^*^), which subsequently reacts with surrounding oxygen to generate singlet oxygen (^1^O_2_^*^). The newly generated ^1^O_2_^*^ then oxidizes available amino acid residues on the tissue surface, forming reactive carbonyl groups that undergo rapid Schiff base reactions with rich amine groups on chitosan,^[^ ^33^, ^34^, ^35^, ^36^ ^]^ resulting in stable covalent bonding (Figure 1b; Figures S1 and S2, Supporting Information). We use silver nanowires (AgNWs) to form a highly conductive layer, which has high optical transparency in the visible light region, showing that AgNWs do not significantly alter the light absorption characteristics of the hydrogel film (Figures S11 and S12, Supporting Information). An encapsulating layer of SEBS (styrene‐ethylene‐butylene‐styrene) was applied to enhance the mechanical robustness of the chitosan film.

The wide applicability of the STICH interface was demonstrated through its strong adhesion to diverse organ surfaces (Figure S4, Supporting Information). Spatially defined photochemical tissue adhesion was achieved by site‐selective green light irradiation (Figure 1c). The covalent bonding enabled by the STICH interface shows strong adhesive capability with a lap‐shear strength of up to 160 kPa (Figure 1d; Figure S3, Supporting Information), higher than PEDOT:PSS‐Based electrical bioadhesive interface,^[^ ^19^ ^]^ chronological adhesive hydrogel patch (CAHP),^[^ ^13^, ^21^, ^22^, ^23^ ^]^ PAA‐NHS based e‐bioadhesive interface,^[^ ^9^ ^]^ photo‐crosslinked adhesive hydrogel,^[^ ^25^ ^]^ and comparable to 3D printable tissue adhesive.^[^ ^20^ ^]^ Furthermore, the silver nanowires endowed the STICH interface with excellent electrical performance, achieving a conductivity of 1000 S cm^−1^, higher than that of previous reported electrical interface^[^ ^9^, ^13^, ^19^, ^21^, ^22^, ^23^, ^24^ ^]^ (Figure S3, Supporting Information). Collectively, these features highlight the unique advantages of the STICH interface in medical applications, enabling spatially programmable and site‐selective adhesion and functionally coupling bioelectronic interfaces during surgical interventions.

### 2.2 Adhesion and Electrochemical Performance

**Figure 2:**
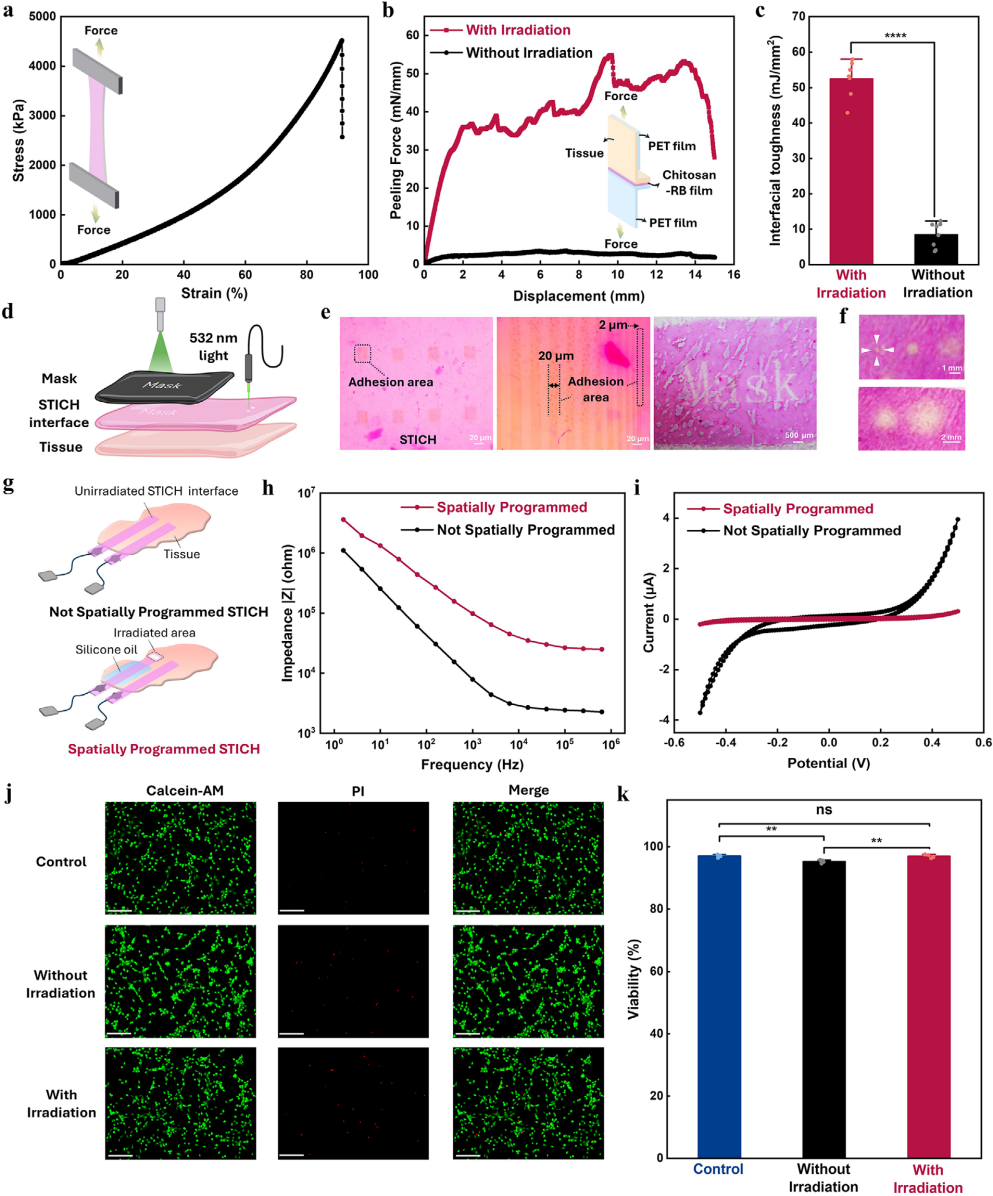
Physical and electrochemical properties of STICH interface. a) The stress–strain curve of STICH interface. b) The T‐peeling adhesion test on tissue. c) The comparison of interfacial toughness without and with irradiation (with irradiation: *n* = 7; Without irradiation: *n* = 8; unpaired two‐sample t‐test; ^****^, *p* < 0.0001). d) Schematic illustration of spatially programmable patterning. e) The patterned grid, linear patterns, and text (left, a grid pattern with 20 µm × 20 µm squares and the dashed line marks the adhesion area; middle, a linear array with a width from 32 to 2 µm; right, the patterned text “mask”). f) The patterned circle array with the direct‐writing method. g) Schematic illustrations of the unirradiated and irradiated STICH interface. h) Electrical impedance spectroscopy (EIS) measurements of STICH without and with light irradiation. i) The cyclic voltammetry (CV) curves of STICH without and with light irradiation. j) Representative live (green) and dead (red) fluorescence images of fibroblasts cultured on a well plate (control) and STICH interface with and without irradiation after 24 h (scale bar, 200 µm). k) Cell viability of control group and STICH interface without and with light irradiation (*n* = 4; one‐way ANOVA test; ns, *p* > 0.05; ^**^, *p* < 0.01).

To further understand the adhesion performance of the STICH interface, we first evaluated its mechanical robustness. STICH can maintain its structural integrity under uniaxial strain of ≈90% (**Figure** **2**a). The high stretchability makes it suitable for integration with mechanically dynamic tissues such as the heart (which undergoes up to ≈60% strain), skeletal muscle, and cerebral cortex.^[^ ^37^, ^38^, ^39^ ^]^ Subsequently, we assessed the interfacial toughness and shear adhesion strength of the STICH interface. The initially dry STICH interface rapidly absorbed interfacial water (Figure S10, Supporting Information) and formed conformal and seamless contact with the tissue surface, which is a prerequisite for covalent bonding. Results from the T‐peeling test demonstrated that the STICH interface, upon activation by green light, forms covalent bonds with tissue surfaces, enabling robust adhesion that resists mechanical separation with tweezers (Figure S6A,B, Supporting Information). The interface exhibited high toughness (≈52.4 mJ mm^−2^) and shear strength (160 kPa) (Figure 2b,c; Figure S9, Supporting Information). Comparable strong adhesion was also observed on various tissue surfaces (Figures S6D and S7B, Supporting Information). In contrast, STICH interfaces without green light exposure, relying on physical interactions, could be easily peeled off (Figures S6C and S7A, Supporting Information), showing substantially lower interfacial toughness (8.5 mJ mm^−2^) and shear strength (45.8 kPa) (Figure 2b,c; Figure S9, Supporting Information). To remove the film without damaging the tissue at the end of its lifetime, sodium bicarbonate solution^[^ ^40^, ^41^ ^]^ can be applied to the adhesive interface for 30 s to 5 min. Bicarbonate solution deprotonates the amine groups on chitosan and subsequently mechanically disintegrates the chitosan layer to realize easy removal of the STICH (Video S3 and Figure S8, Supporting Information).

Bioadhesives, including those using Rose Bengal photochemical reactions,^[^ ^42^, ^43^ ^]^ are prefabricated with printing, moulding, casting, injection, and laser irradiation and lack of interoperative in situ adhesion patterning.^[^ ^9^, ^19^, ^20^, ^21^, ^22^, ^23^, ^24^, ^42^, ^43^, ^44^ ^]^ STICH enabled light‐activated STICH allows adhesion to be confined to microscale regions by situ and direct‐writing technique (Figure 2d). As shown in Figure 2e, the adhesion areas appeared lighter in color due to photobleaching of Rose Bengal, forming grid patterns (20 µm × 20 µm squares) and a linear array (width from 32 to 2 µm), while the non‐adhesive regions remained pink. Furthermore, masked irradiation enabled the clear appearance of the word “Mask” where the adhesion was imprinted on. In addition, the optical fiber can be used for directly writing the patterns (Figure 2f).

We next assessed its electrochemical properties of STICH in vitro. The swelling of STICH upon contact with moist biological surfaces facilitated intimate physical contact. This conformal contact not only promoted subsequent light‐induced adhesion but also effectively minimized both interfacial and contact resistance, yielding a low impedance of 7.86 kΩ at 1000 Hz (Figure 2g,h). To demonstrate site‐selective electrical coupling, we irradiate green light only at the tip of the rectangular‐shaped STICH and use silicone oil as a passivation layer. Without light irradiation, silicone oil can completely insulate the electrode (Figure S14, Supporting Information). However, the spatially programmed STICH, with light irradiation and silicone oil (Figure 2h, red line), yields an impedance of 98.3 kΩ at 1 kHz, which is 13 times higher compared to the sample without patterned light irradiation and silicone oil passivation (Figure 2h, black line). In addition, spatially programmed STICH has substantially different CV with lower electrochemical current compared with samples without spatial programming (Figure 2i; Figure S13A, Supporting Information). Moreover, the charge storage capacity (CSC), a key parameter for electrodes intended for electrical stimulation, remained comparable between the two configurations (Figure S13B, Supporting Information). Furthermore, light irradiation on the STICH interface did not substantially alter the electrochemical properties at the adhesion site, as validated by EIS and CV (Figure S15, Supporting Information). Together, the CV, EIS, and CSC results indicate that only the light‐irradiated tip formed the reliable electrical coupling with the biological tissue.

The STICH interface demonstrated good in vitro biocompatibility. Embryonic fibroblasts cultured on STICH interfaces with green‐light irradiation exhibited no significant difference in viability compared to cells cultured on well plates (Figure 2j,k). Rose Bengal (RB) loaded in the interface without irradiation may gradually diffuse into the culture medium and potentially affect cell viability, which leads to the lower viability of fibroblasts cultured on the STICH interface without irradiation. Nevertheless, cells were able to maintain viability above 95% even in the presence of 0.1 mg mL^−1^ RB in the culture medium (Figure S17, Supporting Information), which is consistent with the previous studies that reported that RB is non‐cytotoxic^[^ ^45^ ^]^ and has been used clinically.^[^ ^46^ ^]^ Furthermore, the cells maintained acceptable viability even after 7 days of in vitro culture (Figure S18, Supporting Information). Together with the good cellular compatibility, the effective removal of silicone oil using safe surfactants during device explantation (Figure S16, Supporting Information) further supports the practical biocompatibility of the STICH interface.

### 2.3 Mechanical Coupling for Directional Electromechanical Sensing

During each cardiac cycle, the myocardium deforms rhythmically in complex, anisotropic patterns, with strain magnitude and direction varying from one region to another.^[^ ^47^ ^]^ When a strain sensor is affixed via a continuous, unpatterned bioadhesive, the tissue motion is homogenized and transmitted uniformly across the entire device, obscuring these local strain differences.^[^ ^48^ ^]^ By spatially patterned adhesion nodes with STICH, we can localize the mechanical load path and couple mechanical strains to the sensor only at light‐defined sites. This spatial programming enables intra‐operative selection of the principal strain axis, allowing the surgeon to monitor a specific strain direction on a curved tissue surface with heterogeneous strains.

**Figure 3:**
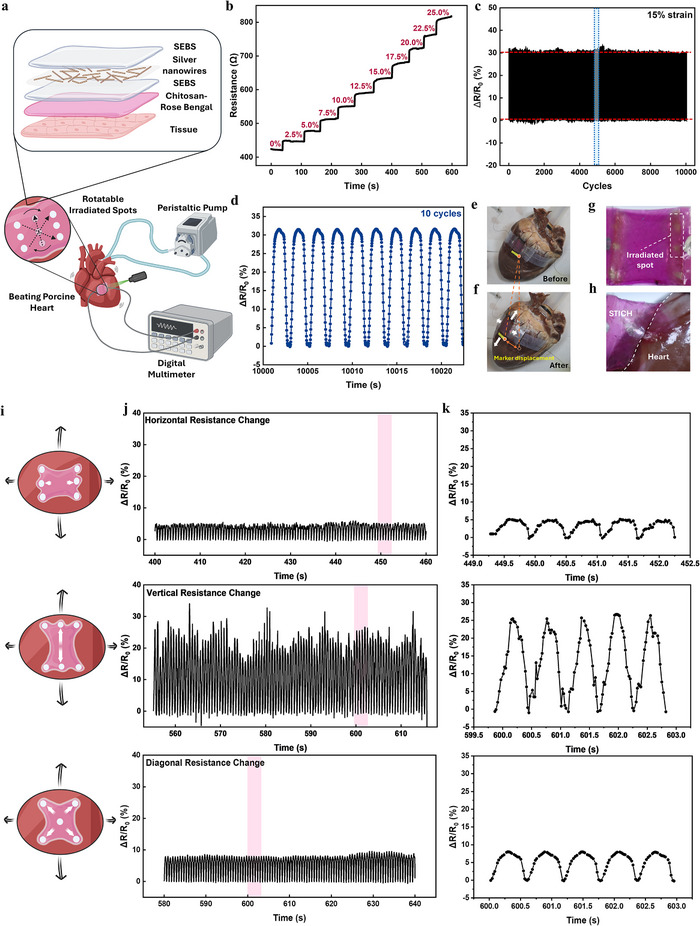
Mechanical coupling for directional electromechanical sensing. a) Schematic illustration of the electromechanical sensing experiment setup and the layer composition of the STICH sensor. b) The resistance changes under different static strains. c) Resistance changes during 10000 strain cycles under 15% strain. d) Resistance changes during 10 strain cycles under 15% strain (the zoom‐in view of the dashed box in c). e,f) The *ex vivo* porcine heart before and after pumping (The orange dashed lines and arrow represent the displacement of the marker, and the white arrow represents the strain directions of the beating heart model). g) The patterns of the STICH interface after irradiation (the dashed box represents the points after being irradiated by light). h) The interface between adhered STICH and the heart surface. i) Schematic illustration of three irradiation patterns for directional electromechanical sensing (the black arrows represent the strain on the heart; the white arrows indicate the directions of strain on STICH; the length of the arrow indicates the magnitude of the strain. j) Representative horizontal, vertical, and diagonal resistance changes‐time curves on *ex vivo* porcine heart model. k) The directional resistance changes during 5 cycles corresponding to the pink area in the j.

To realize directional electromechanical sensing via site‐selective mechanical coupling with tissue, we constructed a strain sensor with a chitosan/RB adhesion layer (**Figure** **3**a). The resulting STICH sensor detects strain through resistance changes in its deformable AgNWs network. It showed a linear resistance increase from 0% strain and 25% strain. (Figure 3b; Figure S19, Supporting Information) During 10000 continuous strain cycles, the sensor maintained a relatively stable sensing performance with low hysteresis. (Figure 3c,d)

We demonstrated spatially patterned adhesion of STICH on an *ex vivo* porcine heart. Pressurized air input was used to mimic heartbeats (Figure 3a), and displacement of surface markers on the porcine heart (Figure 3e,f) indicates volumetric expansion and associated strain changes. By tracking markers, the estimated strain of the porcine heart in horizontal, vertical, and diagonal directions was quantified in the *ex vivo* model (Figure S20 and Video S1, Supporting Information). The light‐irradiated adhesion points on the STICH interface, appearing in lighter color (Figure 3g), formed robust adhesion with the epicardium (Figure 3h).

We further demonstrated directional strain sensing on an *ex vivo* porcine heart, we used three light patterns to form the adhesion sites in horizontal, vertical, and diagonal orientations (Figure 3i). The cardiac tissue has a maximum horizontal strain of 3.3–4.7% and a maximum vertical strain of 7‐8% (Figure S19, Supporting Information). When the array of adhesion sites was arranged vertically (Figure 3i), the STICH sensor was designed to primarily detect periodic horizontal strain (smaller strain on the porcine heart). We observe a reversible resistance change (ΔR/R_0_) of 5% during the cardiac contraction and relaxation phase (Figure 3j,k, top), which corresponds to be ≈4% strain (Figure S19, Supporting Information). The large vertical strain is unable to effectively couple to an unbounded area in the center, allowing for high sensitivity to strain in the horizontal direction. When the array of adhesion sites was arranged horizontally (Figure 3i), the STICH sensor was designed to primarily detect periodic vertical strain (larger strain on the porcine heart). A substantially higher ΔR/R_0_ of 25–27% (Figure 3j,k, middle) was observed, despite it being placed at the same location subject to the same cardiac motion. The ΔR/R_0_ corresponds to a vertical strain of 8.7–10% (Figure S19, Supporting Information). Lastly, the diagonally distributed adhesion sites result in ΔR/R_0_ of ≈8% (Figure 3j,k, bottom), slightly higher than the horizontal orientation but lower than the vertical orientation and consistent with the actual diagonal strain of *ex vivo* heart model (3.5–5.0%, Figure S20, Supporting Information). In the absence of any adhesive anchoring points, STICH weakly bonds to the cardiac surface and is unable to reliably transduce the strain from cardiomyocytes to the device. As a result, we observed substantially smaller changes in resistance with high noise. (Figure S21, Supporting Information). Similarly, the fully bonded STICH sensor without spatial patterning showed limited strain direction sensitivity (Figure S22, Supporting Information).

The directional sensitivity of the STICH sensor stems from the anisotropic mechanical locking effect induced by a spatially programmed linear array of adhesive islands. Consequently, strains vertical to the STICH adhesion array are efficiently transduced into resistance changes, whereas orthogonal strains are largely decoupled. The STICH sensor enables precise, real‐time detection of regional myocardial mechanics with programmable directional sensitivity. Dynamic in situ customization further allows reconfigurable electrical bioadhesive interfacing for intraoperative monitoring and therapy. Based on these advantages, STICH has the potential to be used in several clinical cases. For example, in atrial fibrillation, intraoperative electrophysiological mapping can identify electrical rotors and locate the regions of functional reentry driving abnormal conduction, allowing STICH to swiftly modulate, restore, or block these pathways with spatial precision.

### 2.4 Intraoperative Electrical Coupling In Vivo

**Figure 4:**
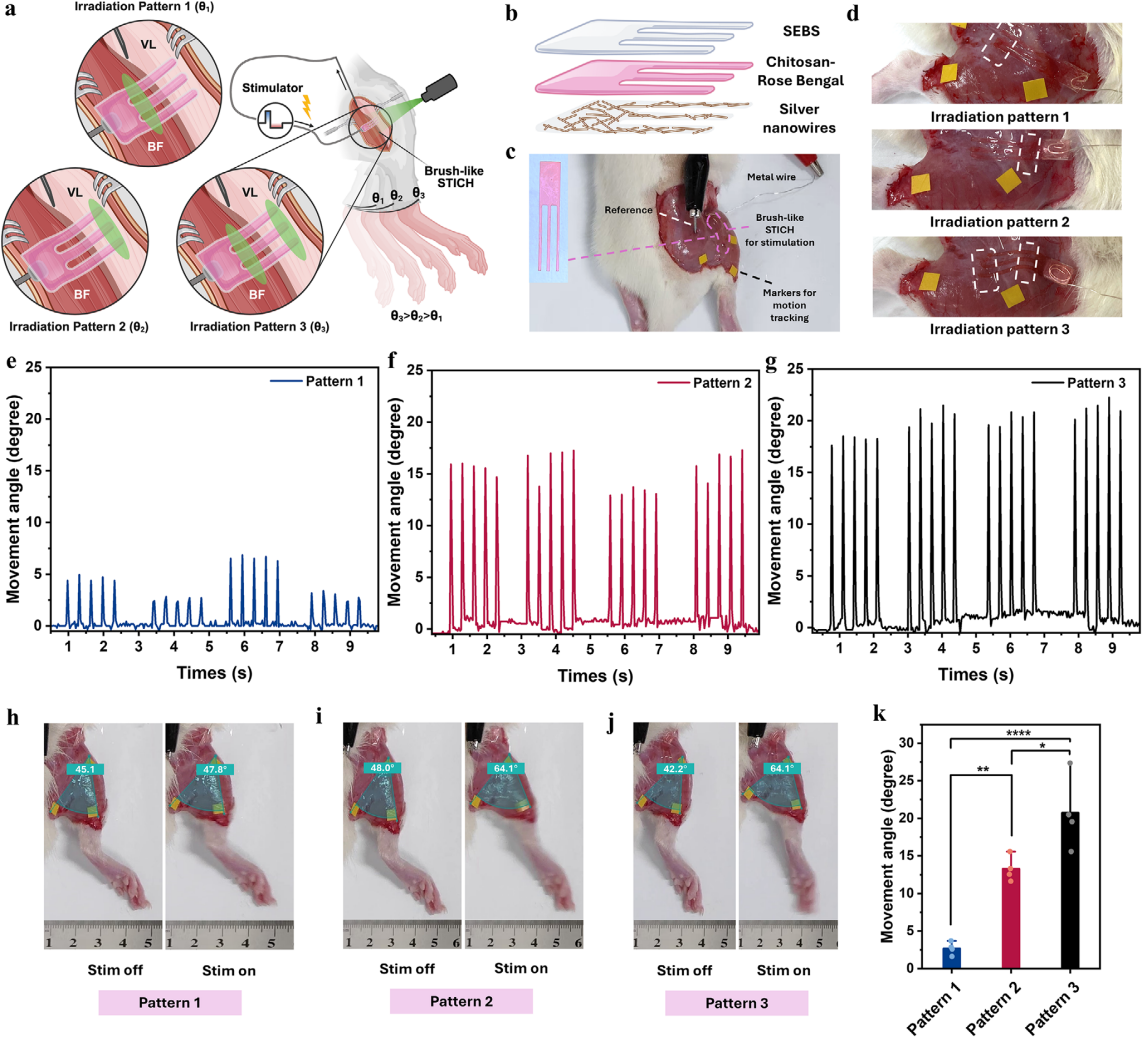
Intraoperative adhesion programming for spatially selective electrical coupling in vivo. a) Schematic illustration of the in vivo intraoperative electrical coupling (BF: biceps femoris, flexor; VL: vastus lateralis, extensor; green ellipse represents the area irradiated by the laser θ_1_, θ_2,_ and θ_3_ represent the movement angle during electrical stimulation following irradiation pattern 1, 2, and 3). b) Schematic illustration of the brush‐like STICH design. c) The configuration of in vivo experiment; Inset: photograph of the brush‐like STICH. d) The photographs of brush‐like STICH with three irradiation patterns (the dashed line marked irradiated area) on muscles. e–g) The angle of leg movements during stimulation with different irradiation patterns. h–j) Representative images of the leg movement in response to electrical stimulation. k) The comparison of angle changes during stimulation following three irradiation patterns (*n* = 4; one‐way ANOVA test; ^****^, *p* < 0.0001; ^**^, *p* <0.01; ^*^, *p* < 0.05).

Next, we demonstrated intraoperative adhesion programming for spatially selective electrical coupling in vivo. STICH was interfaced with the rat muscle for precise electrical stimulation. The biceps femoris (BF) and vastus lateralis (VL) were selected as the target muscle groups, which are both superficial and anatomically adjacent. The BF represents the flexor compartments of the rat hindlimb and is involved in multiple functions (hip extension, thigh abduction, knee flexion), while the VL plays a primary role in the knee extension.^[^ ^49^ ^]^ We positioned a brush‐like STICH interface (**Figure** **4**a) with a three‐layer structure (Figure 4b) on both BF and VL (Figure 4c; Figure S23, Supporting Information). We then used 3 different light patterns (Figure 4d) to selectively form electrical coupling with the 2 muscle groups. Light‐triggered adhesion only at the posterior (Irradiation Pattern 1) and the anterior (Irradiation Pattern 2) of the STICH selectively activated BF or VL, respectively. When both regions were irradiated (Irradiation Pattern 3), strong adhesion formed on both the BF and VL.

When only the flexor (BF) was selectively targeted for specific stimulation (irradiation pattern 1), subtle muscle twitching and a minimal knee joint angular displacement of ≈2.7° were observed (Figure 4e,h and k; Video S2, Supporting Information). Selective stimulation of extensor (VL) alone (irradiation pattern 2) resulted in moderate knee joint movements (Figure 4i; Video S2, Supporting Information), corresponding to a change of 13.3° (Figure 4f–k). Irradiation pattern 3 allows electrical coupling with both flexor (BF) and extensor (VL) muscles simultaneously (Figure 4g). The leg angle increased significantly (Figure 4g; Video S2, Supporting Information) with an average change of 20.7° (Figure 4j,k). When both the flexor and extensor muscles were co‐activated, the resulting forces from two locations have summated to produce the greatest angular displacement in the hindlimb. This phenomenon is due to significant mechanical interactions between neighboring antagonistic muscle groups, primarily resulting from extramuscular myofascial force transmission.^[^ ^50^ ^]^

We evaluated the in vivo biocompatibility of STICH. The immunohistochemical analysis confirmed that neither acute radiation‐induced free radicals nor chronic electrode implantation triggers a significant inflammatory response. Muscle tissue collected 10 min after laser irradiation showed no acute inflammatory response (Figure S24A–C, Supporting Information). Seven days post‐implantation, muscle sections revealed no inflammatory infiltration (Figure S24B, Supporting Information) and a clear collagen fiber structure without pathological remodeling. The basement membrane, interstitium, and other structures are continuous with no signs of rupture or reparative hyperplasia (Figure S24D, Supporting Information). These findings collectively indicate that no significant foreign body reaction or chronic inflammation occurred, and tissue integrity remained intact after irradiation and short‐term exposure to radical species.

By leveraging the site‐selective capability of the STICH interface, we enabled precise electrical stimulation of target muscles to realize the desired functional outcome. Through a single‐channel STICH, we can precisely control leg movement, which previously requires a multiple‐channel stimulator. The STICH interface thus presents a powerful tool for spatially precise electrical stimulation in vivo and for dissecting motor control pathways.

## 3 Conclusion

In this study, we reported a spatially programmable bioadhesive interface (STICH) that enables in situ, microscale resolution site‐selective bioadhesion through light‐triggered covalent bonding with wet biological tissue. It exhibits multi‐organ interface compatibility, mechanical compliance, strong bioadhesion, and favorable electrochemical properties suitable for dynamic physiological environments. STICH serves as a versatile platform for both mechanical and electrical coupling. STICH sensors allow for direction‐sensitive strain detection on ex vivo hearts via in situ, customizable adhesion islands and enable precise in vivo neuromuscular electrical stimulation with intraoperatively defined target regions. By unifying in situ precision patterning, robust adhesion, and low‐impedance electrical interfacing, STICH demonstrates potential as a biointerface that supports future applications in next‐generation, reconfigurable, and patient‐specific bioelectronic medicine.

## 4 Experimental Section

### Materials

Rose Bengal, chitosan, toluene, and isopropyl alcohol (IPA) were obtained from Sigma–Aldrich. Silver nanowire (AgNWs) (*D* = 30 nm, *L* = 20 µm, 10 mg mL^−1^) was obtained from Nanjing XFNANO Materials Tech Co., Ltd. Styrene ethylene butylene styrene (SEBS) (TUFTEC H1062 Grades) was obtained from Asahi Kasei Corporation. Silicone oil was obtained from SG Labware Pte Ltd. DI water was filtered through a Milli‐Q water purification system and had a resistivity of 18.2 MΩ cm at 25 °C.

### Fabrication of STICH for Bench‐top Ex‐Vivo Heart Model Test

The preparation of the chitosan‐Rose Bengal solution followed a previously reported method.^[^ ^30^ ^]^ An AgNWs solution for spin‐coating was prepared by diluting 2 mL of the original AgNWs dispersion (10 mg mL^−1^ in water) with 8 mL of isopropyl alcohol (IPA), resulting in a final concentration of 2 mg mL^−1^ (water: IPA = −11:4 v/v).

Initially, 1.5 mL of the chitosan‐Rose Bengal solution was drop‐casted on a glass slide (2.5 × 7.5 cm) and dried for 2 h at 70 °C in an oven. Subsequently, SEBS solution (0.15 g mL^−1^ in toluene) was spin‐coated onto chitosan‐Rose Bengal film at 1500 rpm for 60 s and allowed to dry overnight at RT to ensure thorough solvent evaporation. In parallel, another glass slide (2.5 × 7.5 cm) was spin‐coated with SEBS solution with the same parameters. After complete drying, the second SEBS‐coated glass slide was spin‐coated 5 times with the prepared 2 mg mL^−1^ AgNWs solution at 500 rpm for 60 s per cycle to achieve optimal percolation and electrical conductivity during mechanical stretching. Finally, the two prepared films were laminated together in the sequence of SEBS‐AgNWs‐SEBS‐chitosan‐Rose Bengal, forming the STICH device for mechanoelectrical sensing.

### Fabrication of STICH for rat Neuromuscular Electrical Stimulation

An AgNWs solution was prepared by diluting 2 mL of the original AgNWs dispersion (10 mg mL^−1^ in water) with 8 mL of DI water, resulting in a final concentration of 2 mg mL^−1^. The AgNWs solution was spin‐coated onto an oxygen‐plasma‐treated (150 W, 1 min) glass slide at 1000 rpm for 60 s and then baked at 120 °C for 10 min. Subsequently, 2 mL of the chitosan‐Rose Bengal solution was drop‐casted onto the AgNWs‐coated slide and dried in an oven at 70 °C for 2 h. Afterward, a SEBS solution (0.15 g mL^−1^ in toluene) was spin‐coated onto the dried chitosan‐Rose Bengal layer at 2000 rpm for 30 s and left to dry overnight at RT. After complete drying, the film was laser‐patterned using CO_2_ laser cutter into a brush‐like structure (three strips with 15 mm long and 1 mm wide). Finally, anisotropic conductive film (ACF, 3M9703) tape was used to connect AgNWs with commercial wires and encapsulated with SEBS to form the STICH device for stimulation.

### Characterization with ATR‐FTIR

The chitosan‐Rose Bengal hydrogels were dried, and the fresh dopamine solution was prepared to investigate the chemical reaction that happened at the STICH‐tissue interface. The hydrogel films soaked in dopamine solution before and after light irradiation were characterized by attenuated total reflection‐Fourier transform infrared (ATR‐FTIR) spectroscopy on a Nicolet iS50 FTIR Spectrometer at room temperature. The data was collected based on the average of 32 scans with a signal resolution of 4 cm^−1^ within the range of 4000–400 cm^−1^. The spectrum of the dried hydrogel film without any treatments was used as background.

### Characterization with SEM and UV–Vis

The morphologies of the AgNWs on freeze‐dried chitosan‐Rose Bengal hydrogels were observed by a field‐emission scanning electron microscope (FE‐SEM, Hitachi Regulus 8230). The UV–vis spectra were collected using a UV‐VIS‐NIR spectrophotometer (SHIMADZU UV‐3600).

### Light‐Initiated Bonding of STICH on Tissue

The adhesion of STICH was activated by a diode‐pumped solid‐state laser that was coupled to a multimode optical fiber (Photonitech VD‐III‐L). The laser emitted a power of 400 mW at 532 nm in continuous waves, with a fiber core diameter of 500 µm. The diameter of irradiation spots was 5 mm with 1500 mW cm^−2^ power density.

### Swelling Behavior

The swelling behavior of STICH with and without SEBS was evaluated using a PBS buffer solution. The mass of the STICH sample was weighed as w_0_, and the STICH samples were immersed in PBS solution for different time scales. After wiping the excess solution on the surface of STICH, the mass of the swelled sample was weighted as w_s_ at different times. The swelling ratio was calculated by the following Equation (1)

(1) $$SwellingRatio\left(\%\right)=\frac{\left(w_{s}-w_{0}\right)}{w_{0}}\times 100\%$$

### Tensile Test

The tensile test was conducted using a universal mechanical testing machine (Instron 68SC‐1) at room temperature. The STICH device, fabricated according to the methods for rat neuromuscular electrical stimulation, was prepared into a rectangular shape (30 mm × 5 mm), clamped with an initial gauge length of 10 mm, and tested with a tensile rate of 5 %/s. The Young's Modulus was calculated from the linear elastic region in the stress–strain curve.

### Adhesion Properties

The adhesion properties of STICH to porcine tissue were evaluated using the lap shear test and T‐peeling test with Instron 68SC‐1. Porcine muscle samples, obtained from local supermarkets, were thoroughly rinsed with PBS followed by deionized (DI) water, then sliced into dimensions of 20 mm × 5 mm × 1 mm (length × width × thickness) prior to testing. All STICH devices and muscle slices were adhered to polyethylene terephthalate (PET) films using cyanoacrylate adhesive as a rigid backing to ensure accurate interfacial adhesion measurements.

For both the lap shear test and 180° peeling tests, 20 mm × 5 mm STICH devices were applied onto porcine muscle slices. The experiment groups were irradiated for 5 min over an area of ≈10 mm^2^, whereas the control groups received no irradiation. The loading rate for both tests was 60 mm min^−1^.

For the lap shear test. The lap‐shear strength, *τ* was calculated by the following Equation (2),

(2) $$\tau =\frac{F_{max}}{S}$$

where *F_max_* is the maximum force in the force‐displacement curve, and *S* is the irradiation area on the STICH device.

For the T‐peeling test, the interfacial toughness *G* was calculated by the following Equation (3),

(3) $$G=\frac{F_{steady}}{w}$$

where *F_steady_* is the steady‐state peeling force from the force‐displacement curve in the 180° peeling test, and *w* is the peeling width of the STICH and muscle slice.

### Resistance Under Strain and Cyclic Strain

The electrical resistance of STICH under static strain and cyclic strain was measured using a digital multimeter (Keithley DMM6500). To evaluate resistance changes under static strain, STICH was fabricated into 20 mm × 5 mm shape according to the heart phantom test, then clamped onto Instron 68SC‐1. Samples were stretched incrementally from 0% to 25% strain in steps of 2.5%, holding each step stable for more than 30 s while recording resistance changes. To measure electrical stability as strain sensors, STICH devices underwent cyclic stretching from 0% to 15% strain at a strain rate of 100%/s for 10000 cycles, with continuous resistance recording with DMM6500 digital multimeter.

### Electrochemical Characterization

The specific measurement conditions for STICH conductivity were as follows: The electrical resistance (R) of STICH was measured using a digital multimeter (Keithley DMM6500). STICH was fabricated into a rectangular shape with length (*L* = 30 mm) and width (*W* = 5 mm), and the thickness (*T* = 6‐10 µm) was measured with a micrometer. The electrical conductivity (σ) was calculated by the following Equation (4),

(4) $$\sigma =\frac{L}{W\times T\times R}$$

The value mentioned in the paper corresponds to the entire STICH composite.

The electrochemical impedance spectroscopy (EIS) and cyclic voltammetry (CV) of the STICH device were performed using a potentiostat (Palmsens 4). Two strip STICH devices (30 mm × 5 mm) were put on the tissue surface as the working electrode and counter electrode. The tissue was moistened with an appropriate amount of PBS buffer before the measurements. EIS measurements were carried out using a sine wave frequency range from 1 Hz to 1 MHz and an amplitude of 10 mV. CV was measured within a voltage range from −0.5 to 0.5 V at a scan rate of 0.1 V s^−1^. Charge storage capacity (CSC) was calculated by the following Equation (5),

(5) $$\mathrm{CSC}=\frac{\int \left(\mathrm{curr}\mathrm{ent}\;\mathrm{dens}\mathrm{ity}\;\mathrm{per}\;\mathrm{area}\right)\mathrm{dE}}{2\times \left(\mathrm{scan}\;\mathrm{rate}\right)}$$

### In Vitro Biocompatibility

In vitro biocompatibility tests were conducted using STICH as a substrate for cell culture. Mouse embryonic fibroblasts (3T3) were seeded on STICH devices (with or without irradiation) fixed on 24‐well plates at 5 × 10⁵ and 1 × 10⁴ cells per well for 24‐h and 7‐day cultures, respectively. The cell culture media consisted of DMEM supplemented with 10% v/v fetal bovine serum and 100 U ml^−1^ penicillin‐streptomycin for both the experimental group and blank control group. An additional experimental group was cultured in media containing 0.1 mg mL^−1^ Rose Bengal in 24‐well plates to evaluate the cytotoxicity of Rose Bengal molecules potentially released from the hydrogel to tissues.

Cell viability was assessed using Calcein‐AM (Sigma–Aldrich) and Propidium Iodide (PI, Thermo Fisher Scientific) for LIVE/DEAD staining. A fluorescent microscope (EVOS M7000) was used to image live cells with excitation/emission at 490 nm/515 nm, and dead cells at 493 nm/636 nm, respectively. Cell viability percentages were determined by counting the number of live (green fluorescence) and dead (red fluorescence) cells by using ImageJ (1.8.0). The 70% cell viability threshold was considered as a general reference, as suggested by ISO 10993‐5 guidelines.

### Ex‐Vivo Porcine Heart Model Test

Porcine hearts were sourced from local supermarkets and thoroughly washed with PBS and deionized (DI) water before use. STICH was irradiated in orientations corresponding to the heart deformation directions: vertical (anterior to inferior), horizontal (left ventricle to right ventricle), and diagonal configurations (along lines between left atrium to right ventricle and right atrium to left ventricle). In the vertical configuration, STICH devices were irradiated at three points each (in total 6 points) on the top and bottom sides (≈5 mm^2^ per point) to anchor horizontal elongation and detect vertical deformation. In the horizontal configuration, irradiation occurred at three points each (in total 6 points) on the left and right sides to anchor vertical elongation and monitor horizontal deformation. For the diagonal configuration, irradiation was applied along the diagonal directions (in total 5 points). The excess silicone oil was applied to isolate unirradiated areas. All STICH devices were fabricated into rectangular shapes of 2.5 cm × 2.5 cm and adhered to the right ventricle of the porcine heart. During testing, porcine hearts underwent volume changes induced by a peristaltic pump (Longer L300‐1FS) at 125 mL mi^−1^n and a compressed air gas supply (Video S1, Supporting Information). The STICH devices, irradiated in defined patterns, were continuously stretched, and resistance changes were measured with a digital multimeter (Keithley DMM6500, USA). Device deformation was simultaneously recorded and analyzed using open‐source video measurement tools.

### Pre‐Stimulation Surgeries

The animal protocol was approved by the Institute of Zoology, Chinese Academy of Sciences. The assigned accreditation number is IOZ‐IACUC‐2023‐137.

All animals were group‐housed under standard vivarium conditions, including static caging with tightly controlled ambient temperature (20–26 °C), relative humidity (30%–70%), and a 12‐h light/dark cycle. Animals had ad libitum access to food and water, except during brief food restriction (4–6 h) prior to anesthesia, as detailed below. Veterinary and research personnel monitored animal health and welfare daily.

Rats were deeply anesthetized via intraperitoneal injection of a ketamine‐xylazine solution (ketamine: 80–100 mg k^−1^g; xylazine: 5–15 mg k^−1^g). Anesthetized animals were placed prone (ventral side down) on a thermostatic surgical platform, maintained at 36–37 °C. The depth of anesthesia was monitored periodically through a toe pinch reflex test. A longitudinal skin incision was made on the right thigh to expose the quadriceps muscle, which was continuously moistened with sterile saline to prevent dehydration and tissue damage. The experimental setup consisted of a laser system with an optical fiber, a function generator, and two types of electrodes: a site‐selective brush‐like stimulating electrode (STICH) and a conventional needle electrode (Figure S23, Supporting Information).

### In Vivo Rat Neuromuscular Stimulation

The brush‐like STICH electrode was used. After surgery, the brush‐like device (anode) was positioned at the proximal thigh close to the hip joint. A conventional needle electrode (cathode) was placed at the distal thigh near the knee joint at the junction area of the lateral femoral, superficial gluteal, and semimembranosus muscles. To ensure effective stimulation and minimize interference, the center‐to‐center electrode distance was maintained at ≈12–20 mm, adjusted based on the exposed muscle length (typically 30–40 mm in adult rats). The brush‐like electrode was aligned parallel to the muscle surface.

The adhesive and conductive properties of the brush‐like electrode were activated using green‐light irradiation delivered via an optical fiber (laser wavelength: 532 nm, spot diameter: 5 mm, power density: 1500 mW cm^−^^2^). Each targeted region was irradiated for 1–2 min. After irradiation, a silicone oil and PDMS mixture (mass ratio of PDMS prepolymer, curing agent, and silicone oil: 10:1:1, cured at room temperature for 6–8 h post‐mixing) was applied to electrically isolate non‐irradiated regions.

Electrical stimulation was delivered using a function generator producing single‐phase square‐wave pulses (pulse width: 0.5 ms, stimulus voltage: 1.0 V, frequency:3 Hz). Each stimulation cycle lasted approximately 2.0 s with a 2.0 s interval between cycles. During irradiation and intermediates between stimulation trains, the muscle surface was regularly moistened using sterile cotton swabs soaked in physiological saline to prevent dehydration.

Leg/knee movements were monitored under a digital microscope throughout the stimulation. Markers positioned on the leg were used for precise measurement of limb swing angles. Animals were euthanized immediately after the completion of the experiments.

### HE and Masson's Trichrome Staining

Green light transmitted via optical fiber (laser wavelength: 532 nm, spot diameter: 5 mm, power density: 1500 mW cm^−^^2^) was used to activate the adhesion and conductivity of the electrodes. Each target area was irradiated for 1–2 min. One group of mice had the electrodes left on the muscle for 10 min, while another group had the electrodes left on the muscle for one week. After carefully removing the electrodes, muscle tissue was harvested and sliced perpendicular to the muscle fibers for staining. Both HE and Masson staining were performed simultaneously.

*Statistical Analysis*: All statistical analyses were conducted using OriginPro. All data in the bar charts are presented as mean ± standard deviation. Unpaired two‐sample t‐tests and one‐way ANOVA were employed to assess statistical significance. ImageJ was used to analyze the in vitro biocompatibility and to quantify the percentage of AgNWs‐covered and uncovered areas. In vivo leg movements and device deformation in ex vivo model were monitored using Kinovea software. Statistical significance levels were indicated as follows: *, p < 0.05, **, p < 0.01, ***, p < 0.001, and ****, p < 0.0001.

## Conflict of Interest

The authors declare no conflict of interest.

## Author Contributions

Y.G., Z.L., and G.X. contributed equally to this work. Y.G., Z.L. and and G.X. performed data curation, formal analysis, investigation, methodology, validation, and visualization; Y.G., Z.L., and G.X. contributed equally to this work. Y.G., Z.L., Z.G., and Y.L. wrote the original draft, reviewed and edited the final manuscript. G.Z. also performed data curation, formal analysis, funding acquisition, investigation, methodology, resources acquisition, and validation. Y.X. performed investigation, methodology, and visualization; M.R.P. performed visualization; X.C., J.Y.K., J.Z., and J.H. performed methodology; T.F. and Q.G. acquired resources; Y.L. also performed conceptualization, resources, funding acquisition, project administration, and supervision.

## Supporting information



Supporting Information

Supplemental Video 1

Supplemental Video 2

Supplemental Video 3

## Data Availability

The data that support the findings of this study are available from the corresponding author upon reasonable request.
