# Supplementary material for: Spatially Programmable Electroadhesive Enables In Situ Site‐Selective Functional Coupling
Source: Adv Mater. 2025 Oct 14;37(50):e11039. doi: 10.1002/adma.202511039 (PMC12710562; doi:10.1002/adma.202511039)
Supplement: Supplementary file 1 — Supporting Information [file ADMA-37-e11039-s001.docx]

Supporting Information

Spatially Programmable Electroadhesive Enables in Situ Site-Selective Functional Coupling

*Yuting Guo^1,2,3^, Zhuoming Liang^1,2,3^, Guoshi Xu^4,5,6^, Zhen Gu^7^*, Yuheng Xu^1,2,3^, Michael Raphael Panganiban^1,2,3^, Xue Cai^1,2,3^, Jet Yong Kiat Lim^1,2,3^, Jiguang Zhang^8^, Jingxiu Huang^9^, Tingting Fan^4,5,6^, Qi Gu^4,5,6^ & Yuxin Liu^1,2,3^**

Y. Guo, Z. Liang, Y. Xu, M. R. Panganiban, X. Cai, J.Y.K. Lim and Y. Liu

1, Department of Biomedical Engineering, National University of Singapore, Singapore, 117583, Singapore

2, The N.1 Institute for Health, National University of Singapore, Singapore, 117456, Singapore

3, Institute for Health Innovation and Technology (iHealthtech), National University of Singapore, Singapore, 119276, Singapore

E-mail: lyx@nus.edu.sg

G. Xu, T. Fan and Q. Gu

4, Human Organ Physiopathology Emulation System, State Key Laboratory of Organ Regeneration and Reconstruction, Institute of Zoology, Chinese Academy of Sciences, Chaoyang District, Beijing, 100101, China

5, Beijing Institute for Stem Cell and Regenerative Medicine, Chaoyang District, Beijing, 100101, China

6, University of Chinese Academy of Sciences, Huairou District, Beijing, 100049, China

Z.Gu

7, School of Chemistry and Biological Engineering, University of Science and Technology Beijing, Beijing, 100083, China

E-mail: guzhen@ustb.edu.cn

J. Zhang

8, Department of Chemical and Biomolecular Engineering, National University of Singapore, Singapore, 117585, Republic of Singapore

J. Huang

9, Department of Anesthesiology, State Key Laboratory of Oncology in South China, Guangdong Provincial Clinical Research Center for Cancer, Sun Yat-sen University Cancer Center, Guangzhou, 510060, China.

Yuting Guo, Zhuoming Liang, and Guoshi Xu contributed equally to this work.

**This PDF file includes:**

**Supplementary figures S1 to S24:**

Figure S1. Reaction mechanism of chitosan-Rose Bengal hydrogel for tissue adhesion triggered by green-light irradiation.

Figure S2. The FTIR spectra of chitosan-Rose Bengal film before and after light irradiation.

Figure S3. Comparison of lap-shear strength and conductivity with previously reported adhesive interfaces.

Figure S4. Robust adhesion of STICH on different organs.

Figure S5. Photobleaching of Rose Bengal on brain tissue.

Figure S6. Adhesion performance of STICH before and after green-light irradiation.

Figure S7. Adhesion performance of STICH devices on kidney.

Figure S8. Film detachment with NaHCO_3_ treatment.

Figure S9. Lap shear test evaluating the lap-shear strength of STICH on porcine tissue.

Figure S10. Swelling behavior of chitosan-Rose Bengal hydrogel in PBS for 120 min.

Figure S11. UV–Vis absorption spectra of chitosan–Rose Bengal hydrogel films with and without SEBS coating.

Figure S12. SEM images of AgNWs percolation networks on STICH.

Figure S13. Effect of spatial programming on voltametric charge and CSC of STICH devices.

Figure S14. Insulation effect of silicone oil.

Figure S15. Electrochemical properties of STICH before and after irradiation.

Figure S16. Electrical impedance spectroscopy (EIS) measurements for studying silicone oil removal efficiency.

Figure S17. In vitro biocompatibility assessment of STICH before irradiation and addition of Rose Bengal.

Figure S18. In vitro biocompatibility assessment of STICH under different conditions for 7 days.

Figure S19. Relative resistance change (ΔR/R₀) of the STICH device under horizontal and vertical tensile strain.

Figure S20. Quantification of heart deformation along three anatomical directions based on video analysis.

Figure S21. Representative resistance changes-time curve of not spatially programmed STICH on *ex-vivo* porcine heart model.

Figure S22. Resistance changes of fully bonded STICH on *ex-vivo* porcine heart model.

Figure S23. *In vivo* experimental setup for muscle stimulation using the STICH device.

Figure S24. H&E staining and Masson’s trichrome staining of STICH interface.

**References**

**Captions for supplementary videos S1 and S2, S3**

**Other supplementary materials for this manuscript include the following:**

**Supplementary videos S1 and S2, S3**

**Supplementary Figures**

**
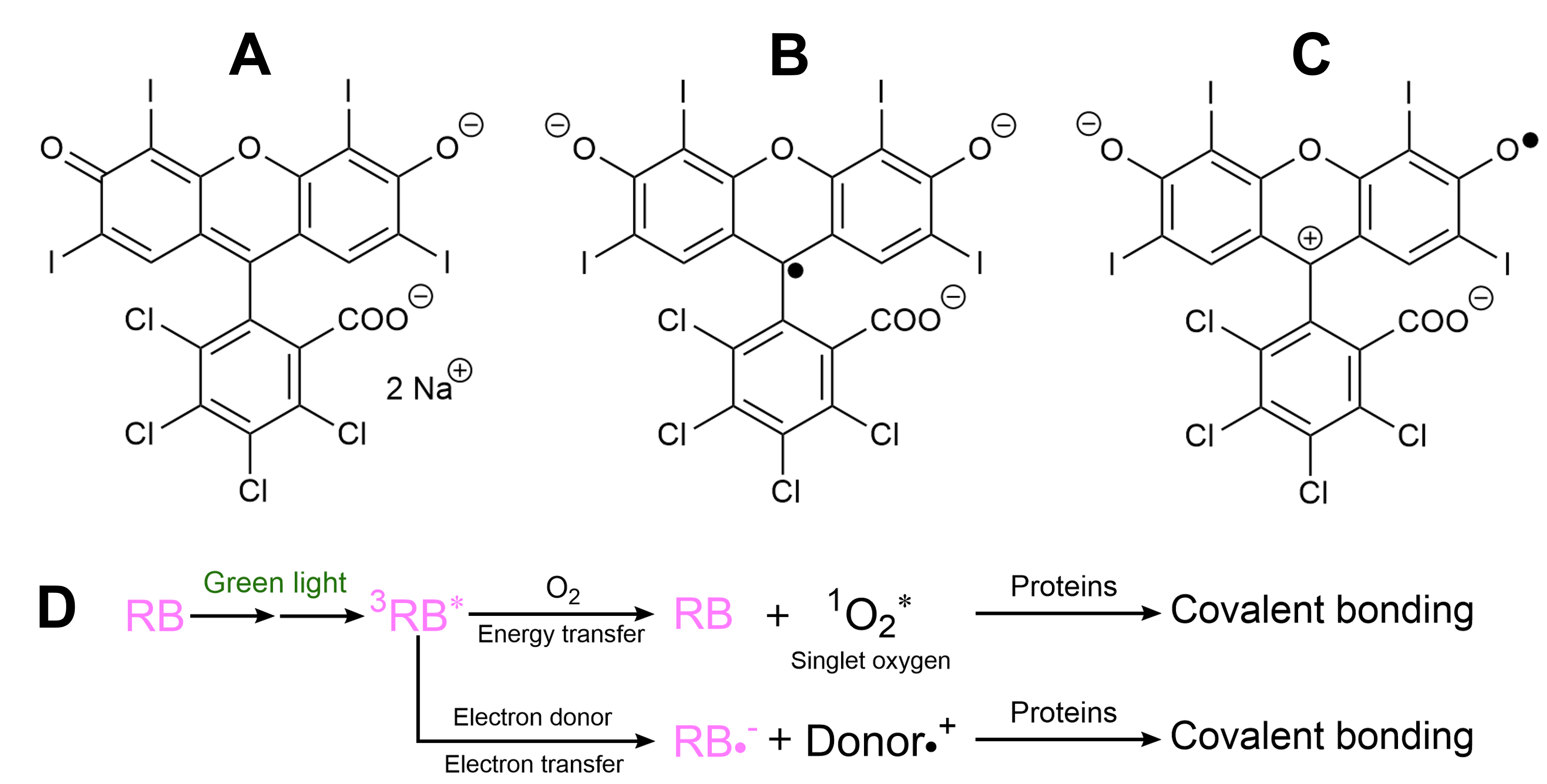
**

**Figure S1. Reaction mechanism of chitosan-Rose Bengal hydrogel for tissue adhesion triggered by green-light irradiation.** Structure of Rose Bengal (A), its reduced form (^3^RB*) (B), its oxidized form (RB·^-^) (C), and the two photochemical pathways for formation of protein crosslinks after green light absorption (D). Energy transfer pathway domain in the presence of oxygen: energy is transferred to oxygen for generating singlet oxygen that further reacts with proteins. Specifically, the Rose Bengal-mediated photochemical reaction at the STICH-tissue interface proceeds via singlet oxygen-induced oxidation of reactive amino acid residues on the tissue surface to aldehyde or ketone groups, which subsequently form imine bonds with amine groups from chitosan through Schiff base reactions.^[1–3]^ RB-photosensitized crosslinking also occurs in the absence of oxygen via amino acids, such as arginine and lysine as electron donors.^[4]^

**
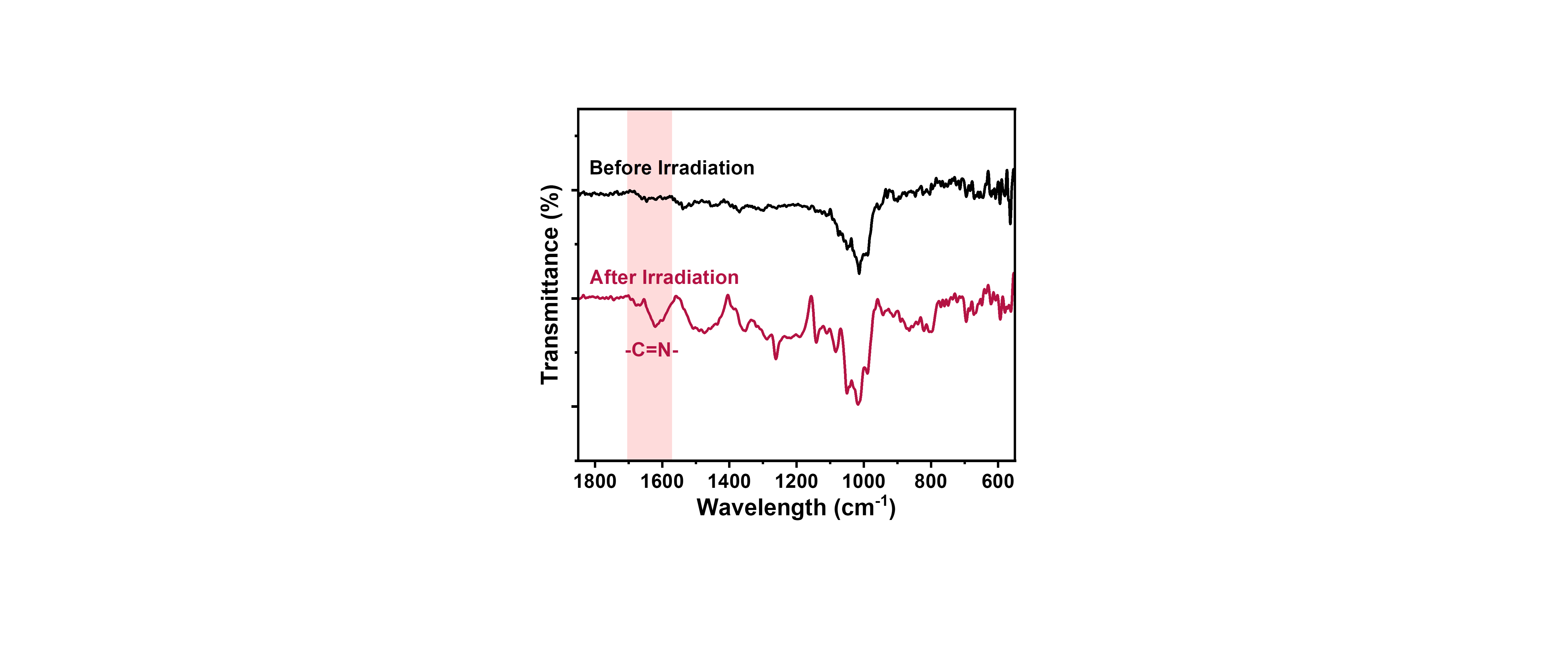
**

**Figure S2. The FTIR spectra of chitosan-Rose Bengal film before and after light irradiation.** The ATR-FTIR analysis on chitosan-Rose Bengal films before and after light irradiation was performed. A new peak corresponding to imine bonds (-C=N-, 1600-1650 cm⁻¹) appeared in the spectra after irradiation, confirming Schiff base reaction happened between o-quinone intermediates and chitosan amine groups.


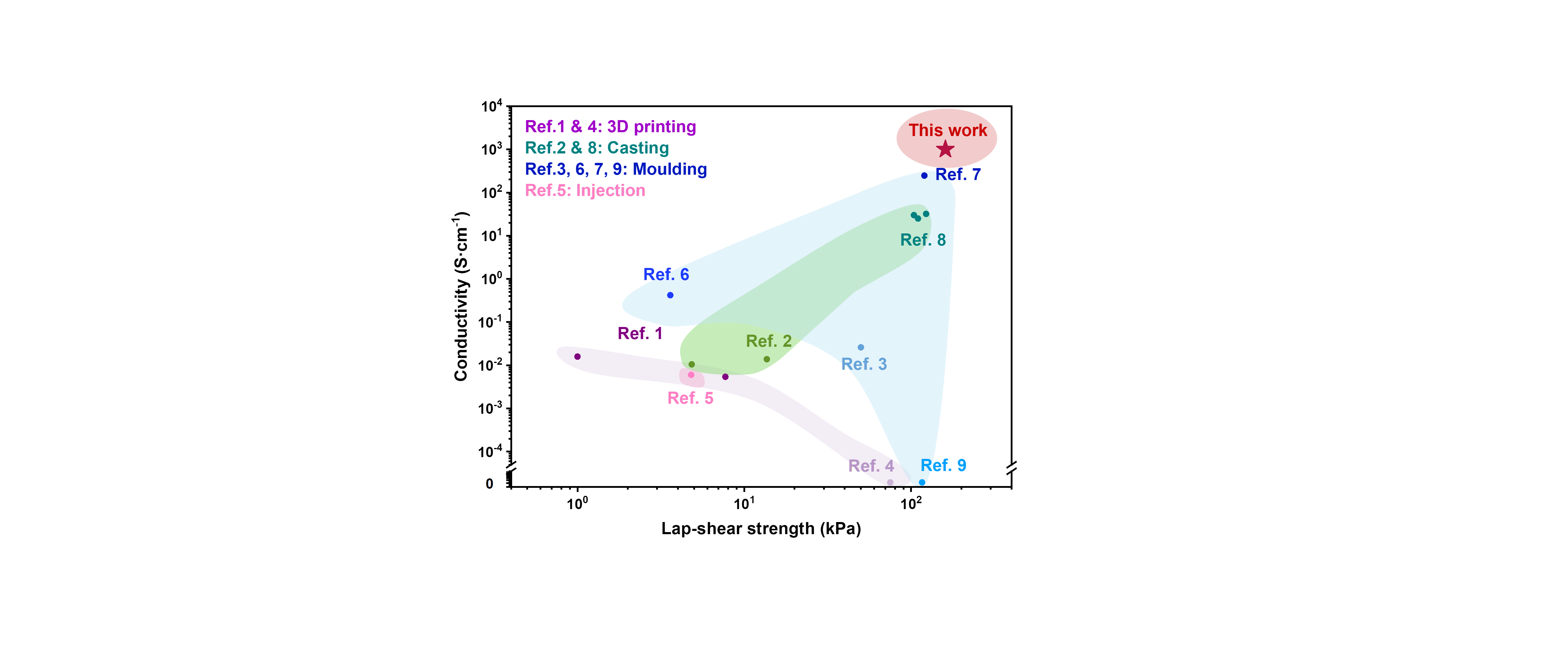


**Figure S3. Comparison of lap-shear strength and conductivity of bioadhesive interface reported in references and this work. (**Ref. 1,^[5]^ Ref. 2,^[6]^ Ref.3, ^[7]^ Ref. 4,^[8]^ Ref.5,^[9]^ Ref.6,^[10]^ Ref.7,^[11]^ Ref.8 ^[12]^, Ref.9 ^[13]^**)**


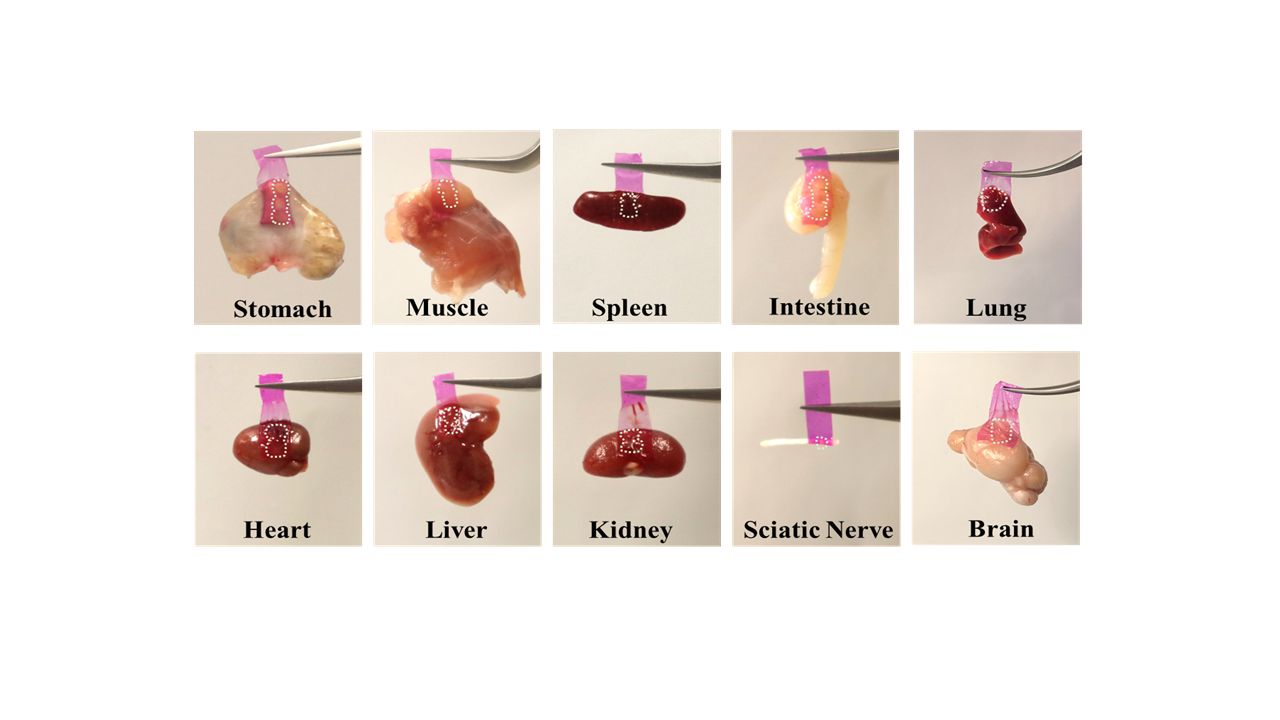


**Figure S4. Robust adhesion of STICH on different organs.** Dashed line marks the irradiation area.


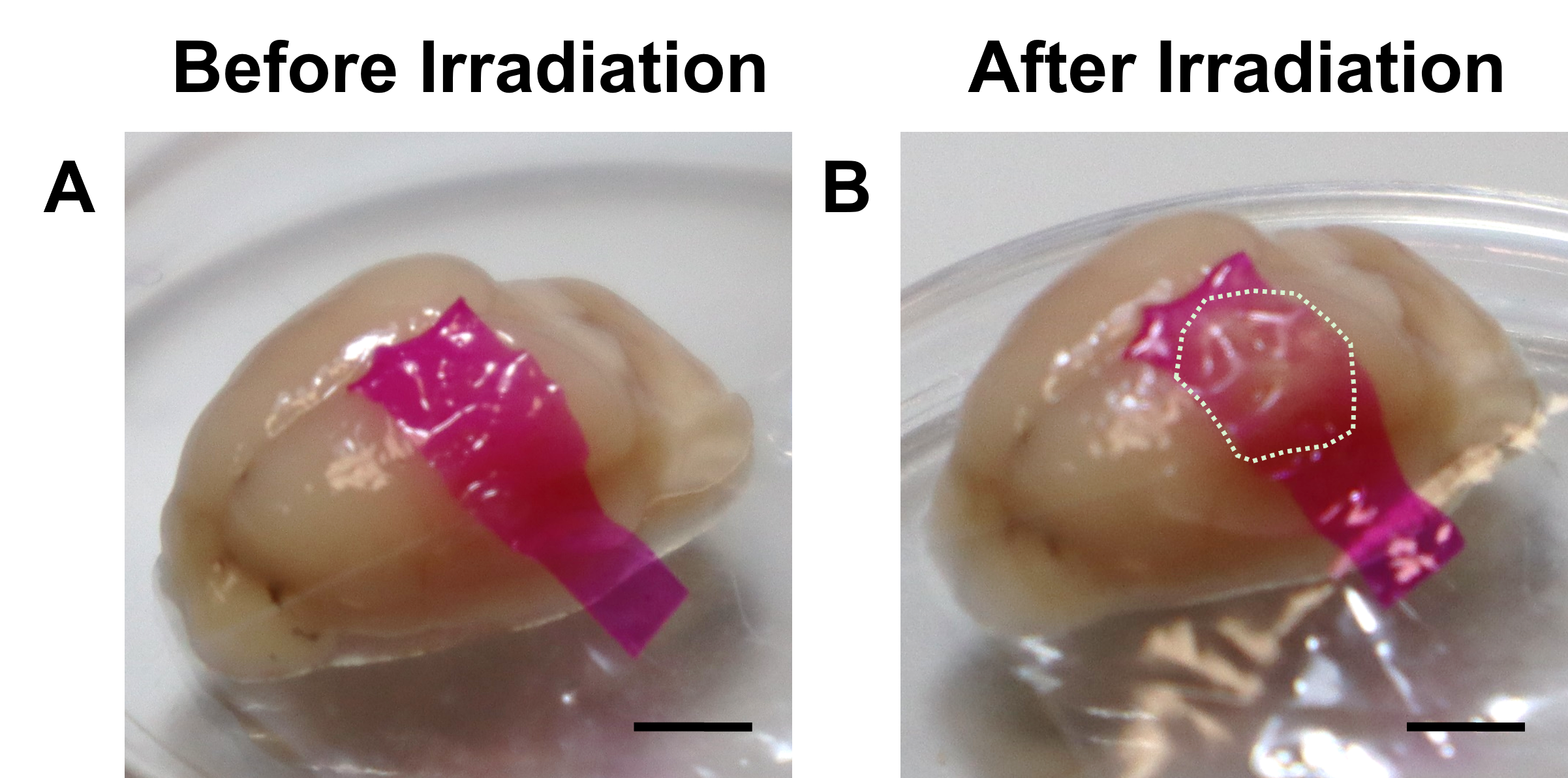


**Figure S5.** **Photobleaching of Rose Bengal on brain tissue.** Images of STICH device on the surface of brain tissue before (A) and after (B) green-light irradiation. The visible photobleaching after irradiation indicates the occurrence of a photo-triggered adhesion reaction between STICH device and the tissue. Scale bars: 5 mm.


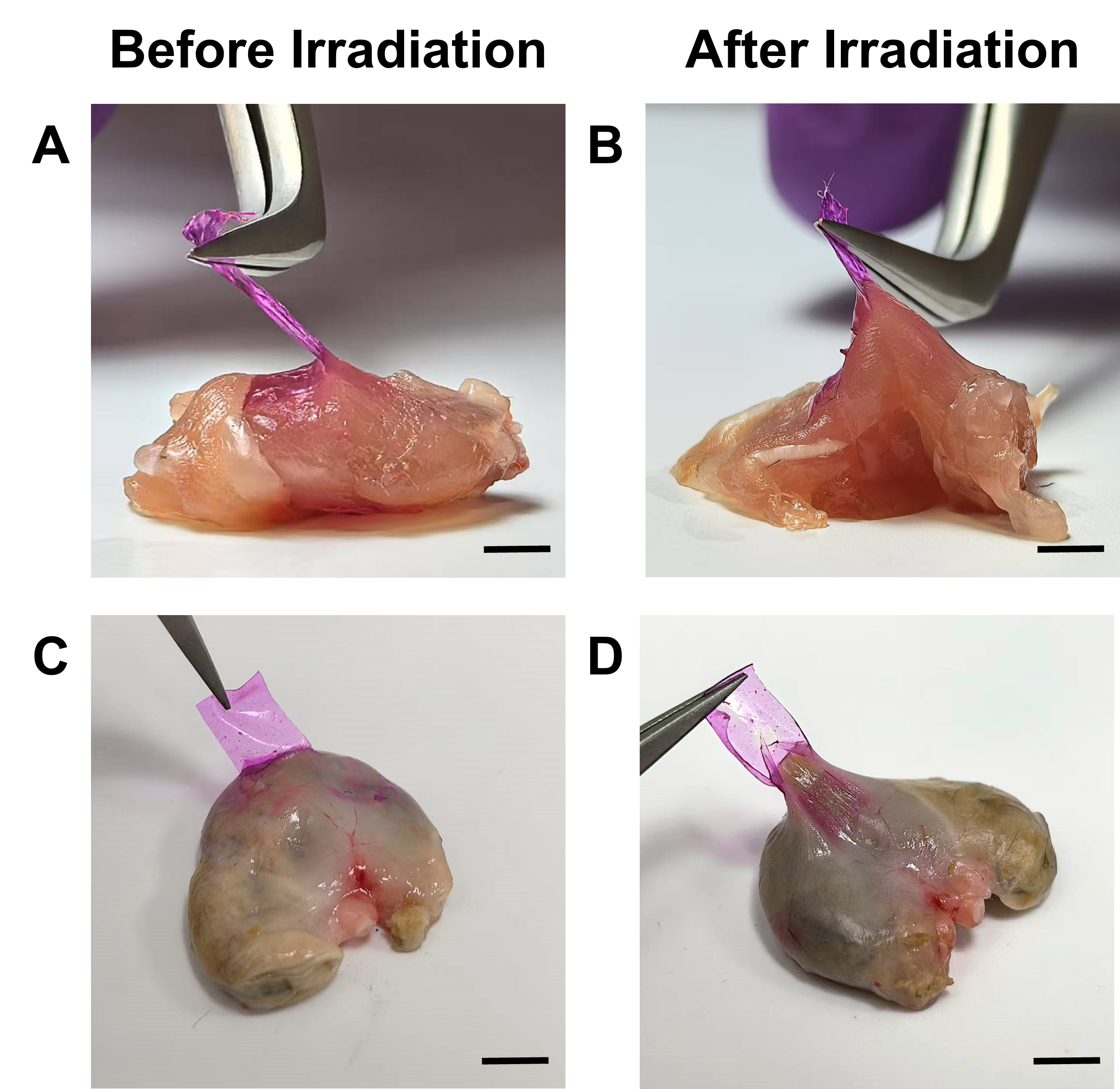


**Figure S6. Adhesion performance of STICH before and after green-light irradiation.** Images of STICH devices being peeled from (A) muscle before irradiation, (B) muscle after irradiation, (C) stomach before irradiation, and (D) stomach after irradiation. Scale bars: 5 mm.


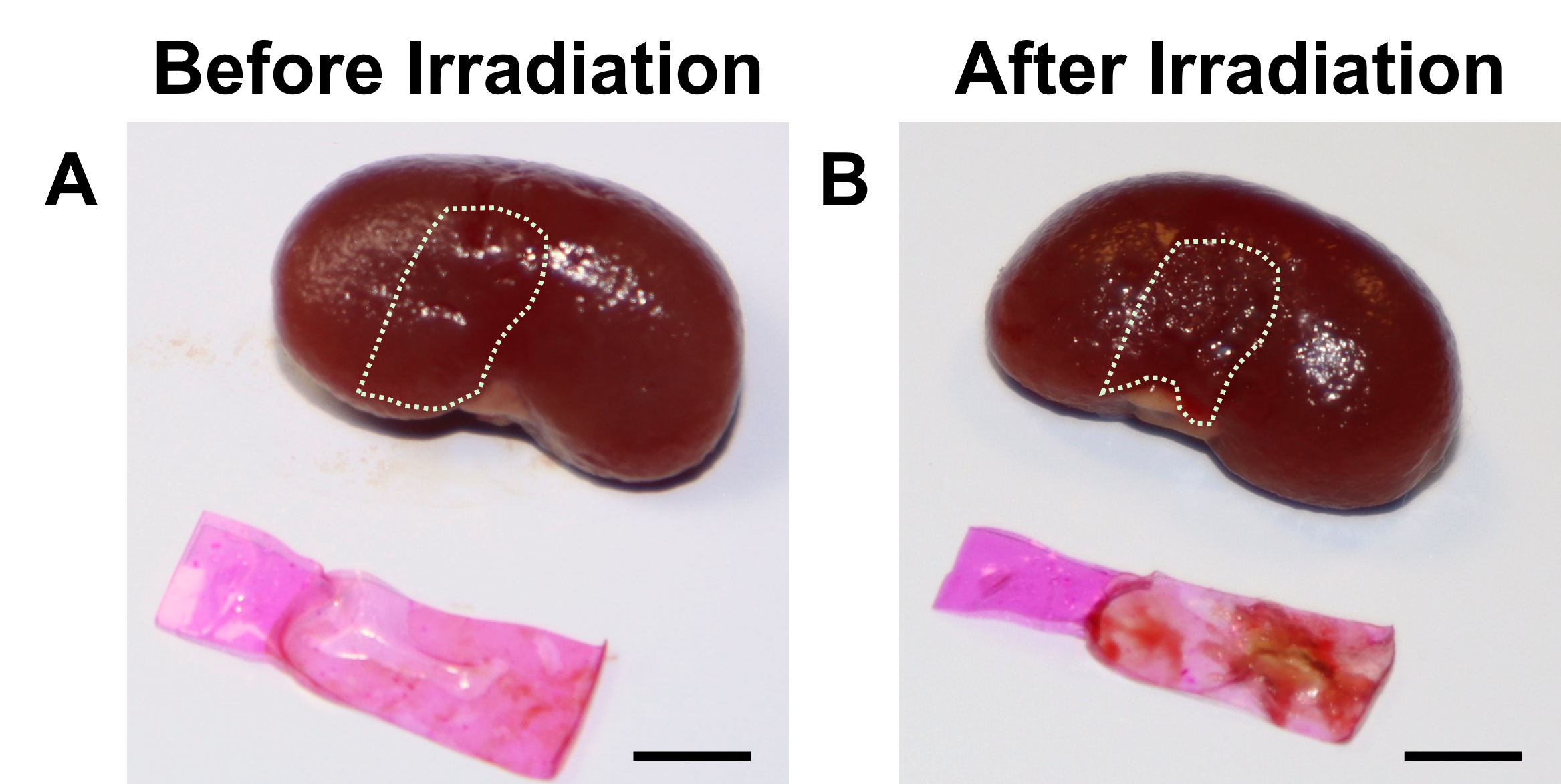


**Figure S7. Adhesion performance of STICH devices on kidney.** STICH devices peeled off from (A) non-irradiated and (B) irradiated areas. Tissue residues clearly remain attached to the device following removal from the irradiated region, demonstrating strong covalent bonding with tissue. Scale bars: 5 mm.


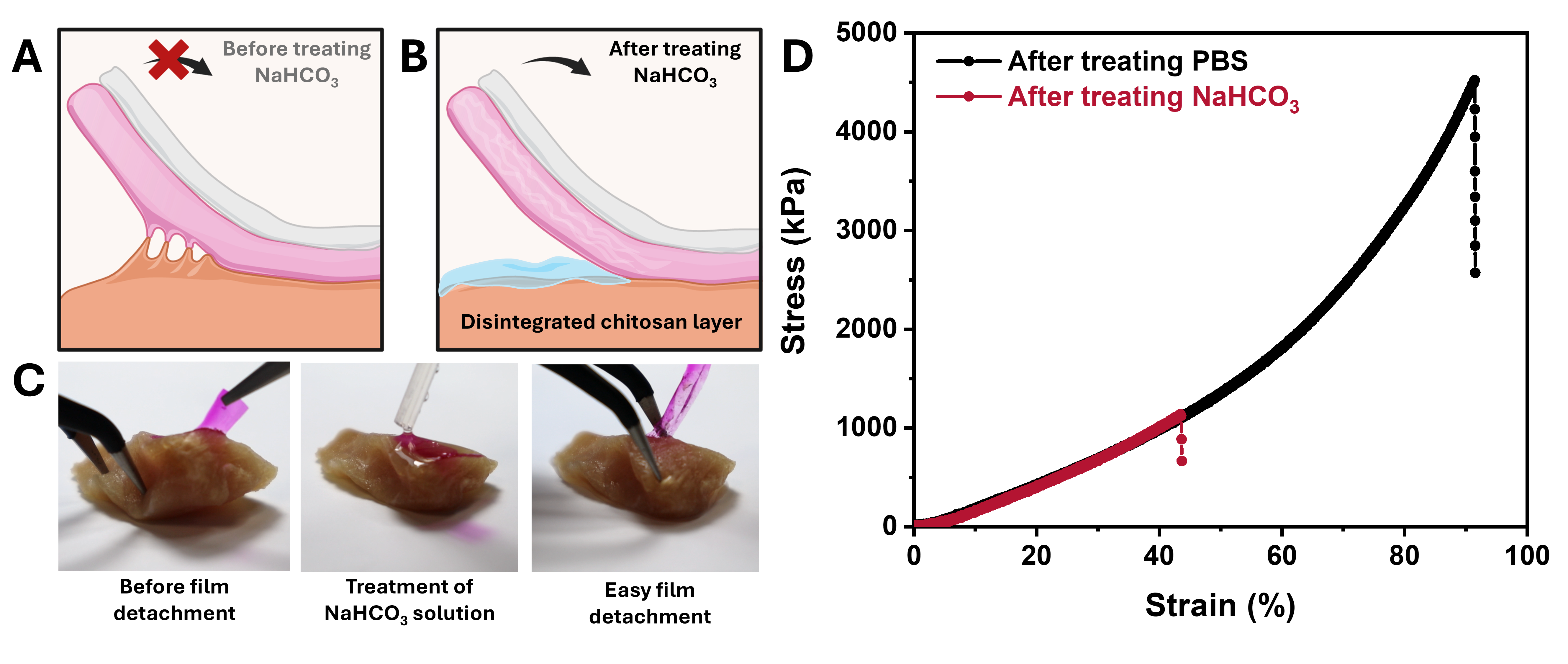


**Figure S8. Film detachment with NaHCO_3_ treatment.** (A) The effective adhesion of chitosan-Rose Bengal film. (B) The film detachment induced by the structural integration of the chitosan layer. (C) The photographs show the steps of film detachment. (D) The comparison of mechanical properties of chitosan-Rose Bengal film after treating PBS and NaHCO_3_. The mechanical properties of bulk chitosan film have been altered, which contributes to the disintegration of entire film.


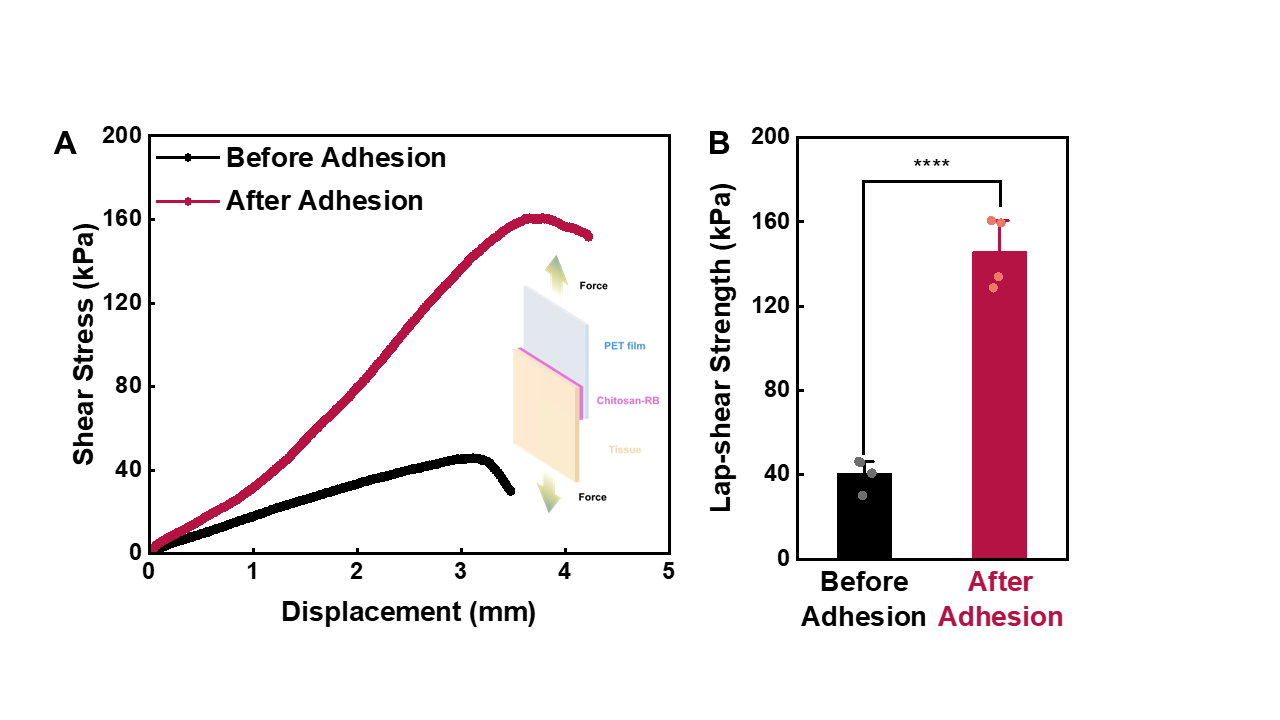


**Figure S9. Lap shear test evaluating the lap-shear strength of STICH on porcine tissue.** (A) Stress–displacement curve. (B) Lap-shear strength (mean ± SD, n = 4) of the STICH device bonded to porcine muscle. (unpaired two-samples t-test; ****, p < 0.0001).


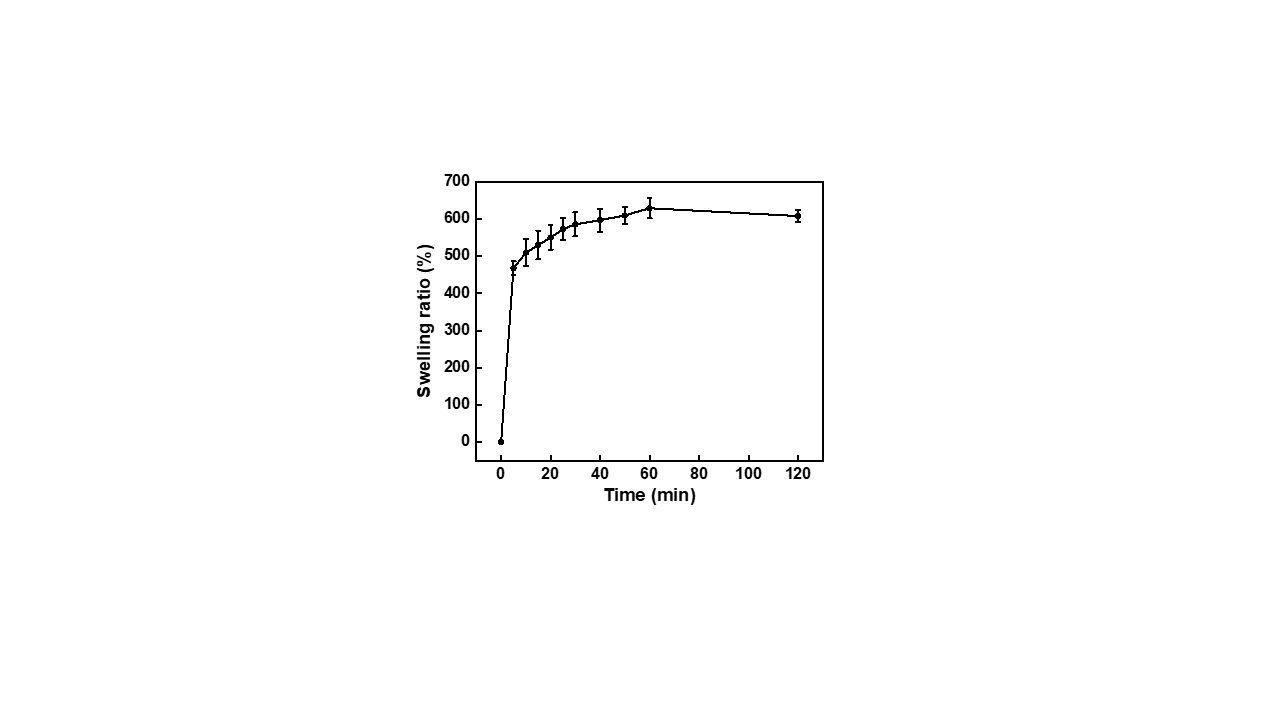


**Figure S10. Swelling behavior of chitosan-Rose Bengal hydrogel in PBS for 120 min.** Swelling ratio (mean ± SD, n = 4) of chitosan-RB hydrogel increase significantly within 120 min.


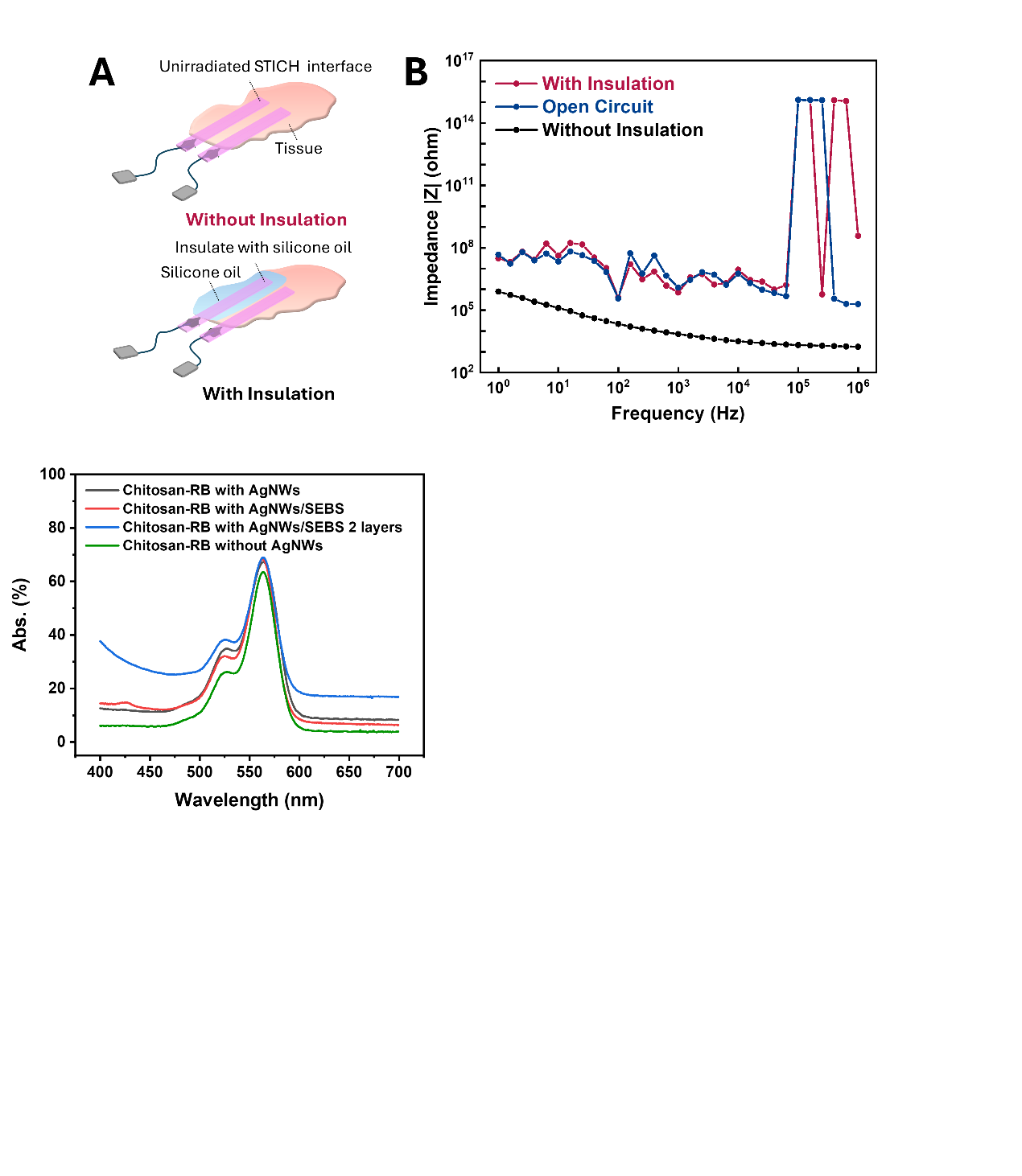


**Figure S11. UV-Vis absorption spectra of chitosan-Rose Bengal hydrogel films.** Absorbance spectra of (i) Chitosan-Rose Bengal hydrogel with AgNWs (black), (ii) hydrogel with AgNWs and a single spin-coated SEBS layer (red, for *in vivo* demonstration), and (iii) hydrogel with AgNWs and double spin-coated SEBS layers (blue, for *ex vivo* demonstration), (iv) hydrogel without AgNWs (green). The consistent absorption peaks across all samples indicate that the addition of SEBS layers and AgNWs do not substantially alter the light absorption characteristics of the hydrogel film.


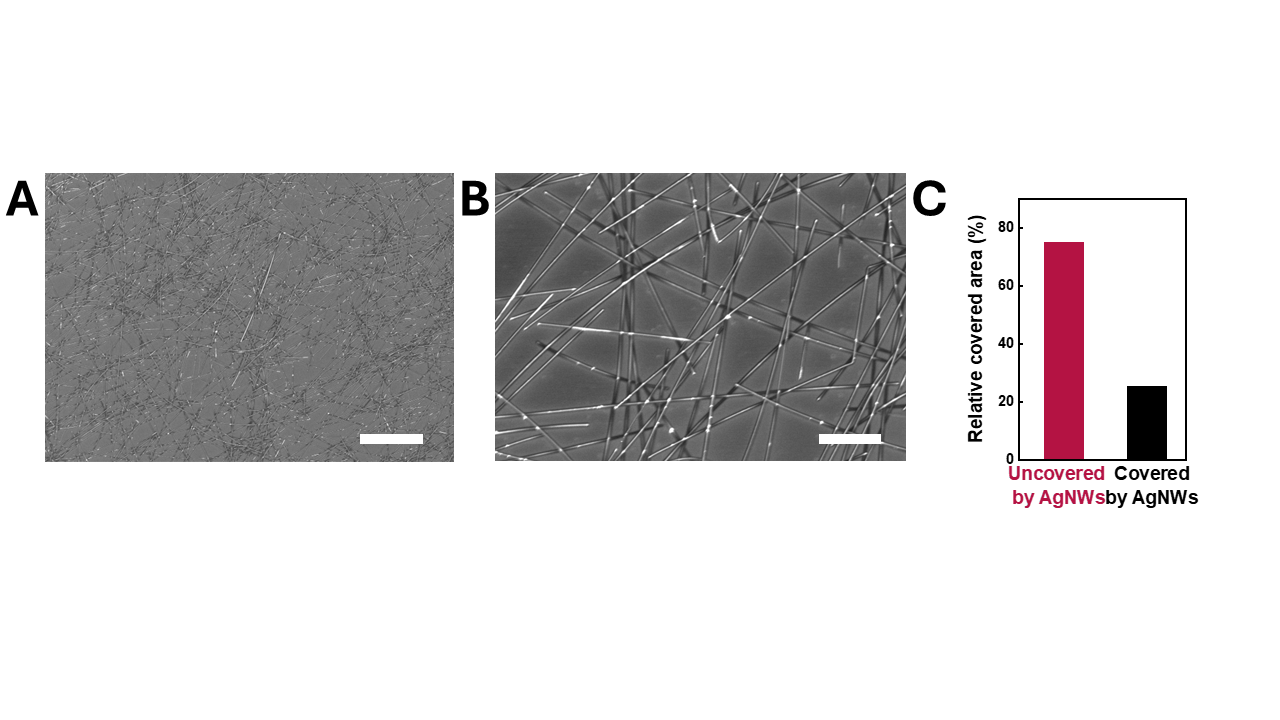


**Figure S12. SEM images of AgNWs percolation networks on STICH.** 2,000×(A) and 10,000× (B) SEM images showing uniform AgNWs distribution on the hydrogel surface, enabling effective electrical interfacing with tissue for stimulation and AgNWs and chitosan-Rose Bengal form percolation. Scale bars: (A) 10 μm, (B) 2 μm. (C) The percentage of area uncovered and covered by AgNWs.


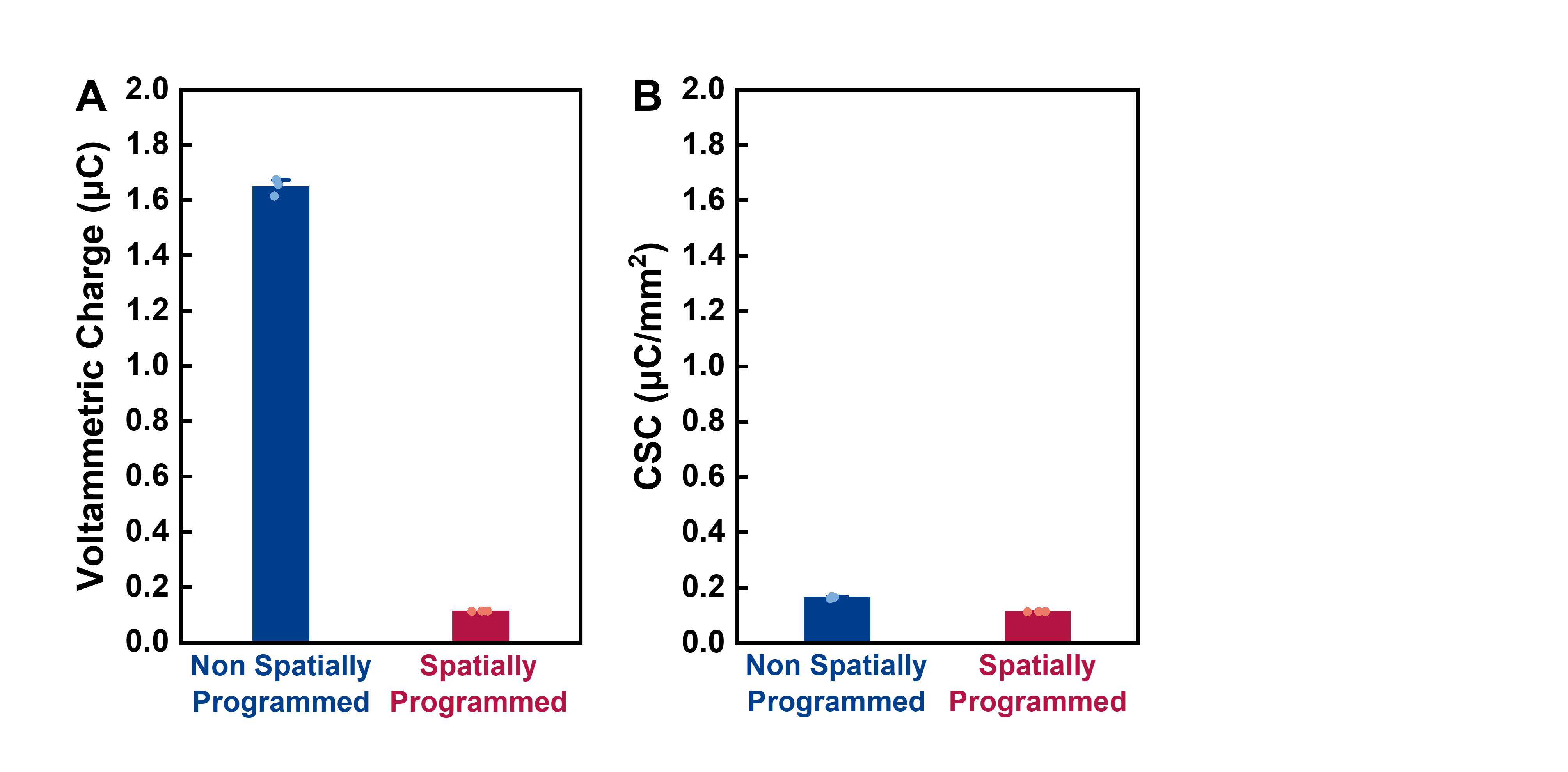


**Figure S13. Effect of spatial programming on voltametric charge and CSC of STICH devices.** (A) Voltammetric charge (mean ± SD, n = 3) for non-spatial programmed (fully interfacing with tissue) and spatially programmed (selective area adhered to tissue, the rest are insulated by silicone oil) STICH devices. (B) CSC (mean ± SD, n = 3) for non-spatially programmed and spatially programmed STICH devices.


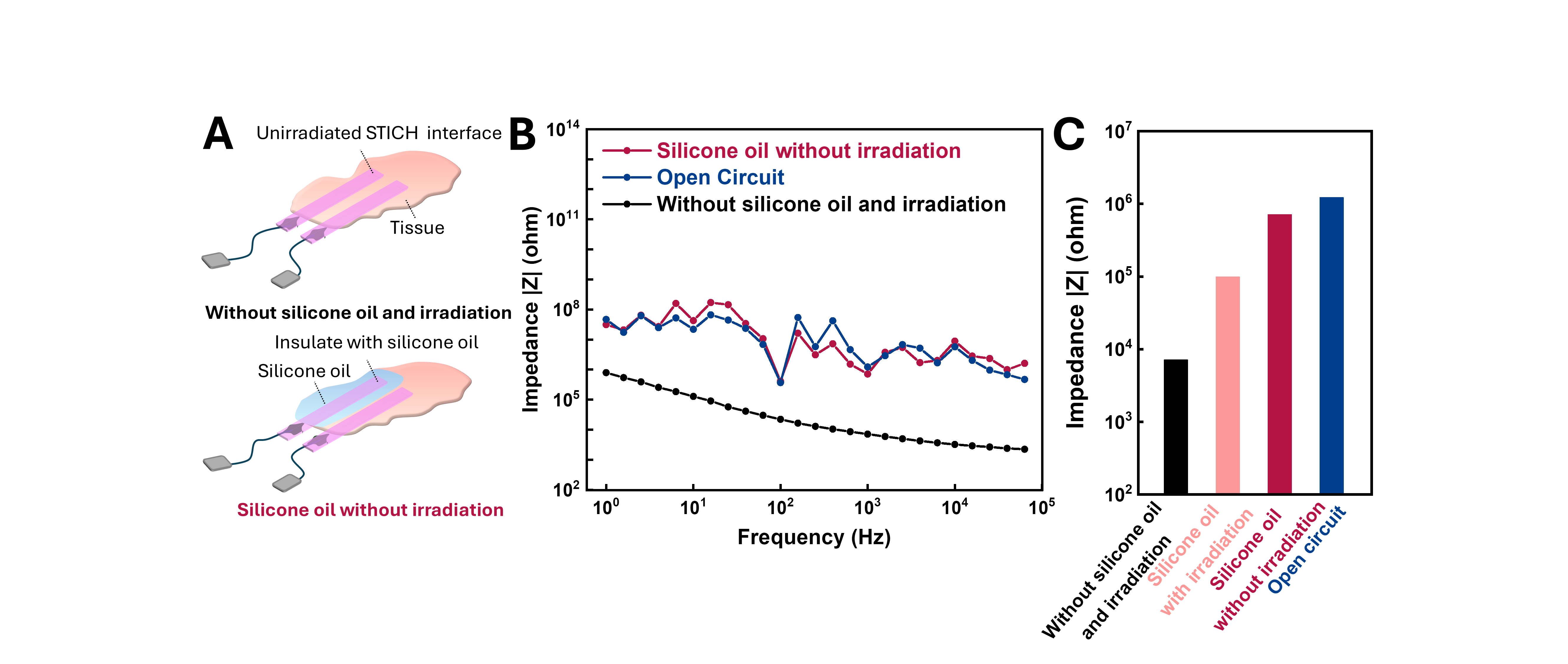


**Figure S14. Insulation effect of silicone oil.** (A) Schematics of STICH interface without and with silicone oil insulation. (B) Electrical impedance spectroscopy (EIS) measurements of STICH without and with silicone oil insulation. (C) Impedance at 1kHz for different conditions: silicone oil without irradiation, silicone oil with irradiation, without silicone oil and irradiation and open circuit.

*
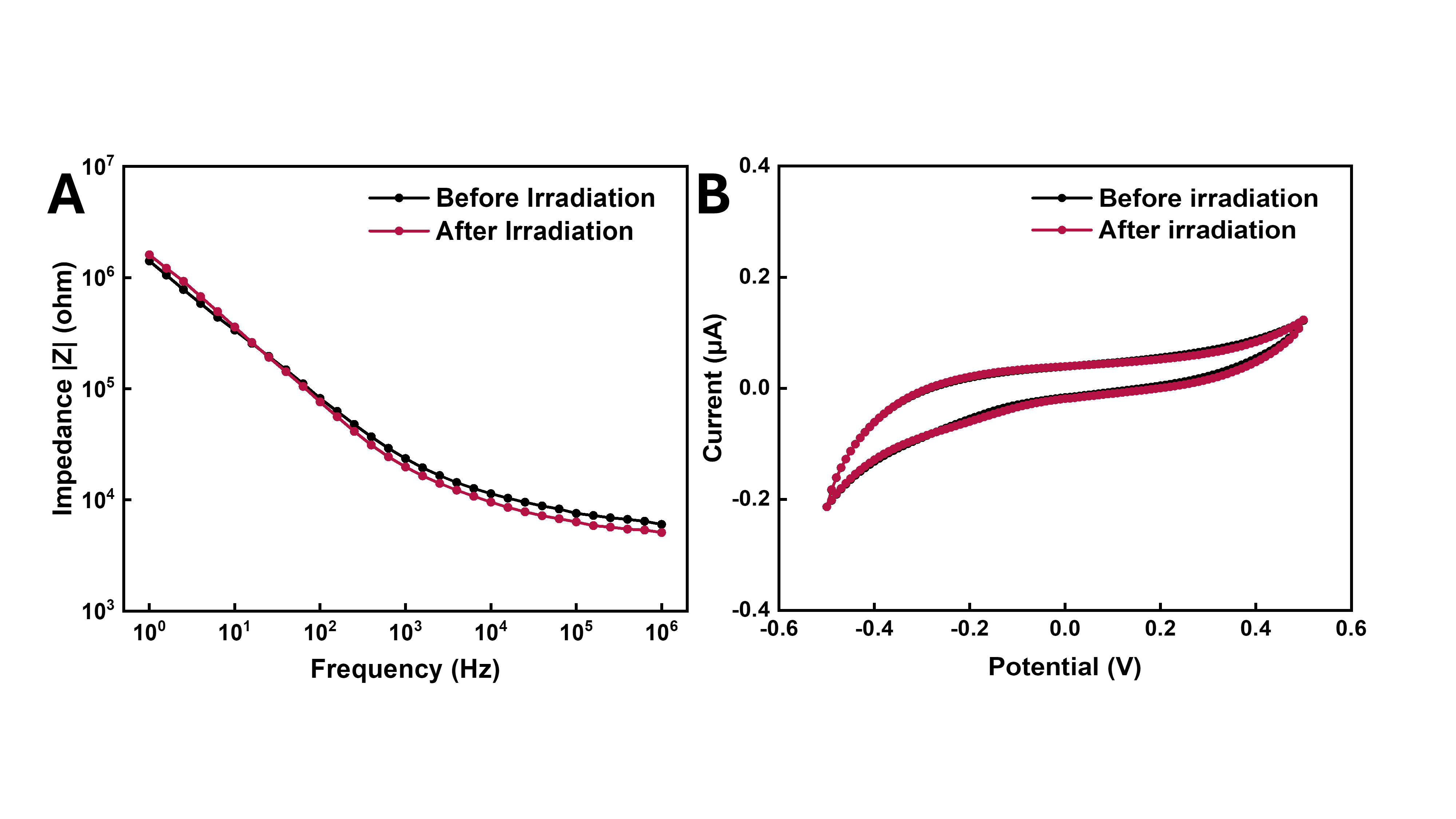
*

**Figure S15. Electrochemical properties of STICH before and after irradiation.** (A) Electrical impedance spectroscopy (EIS) measurements of STICH before and after light irradiation. (B) The cyclic voltammetry (CV) of STICH before and after light irradiation.


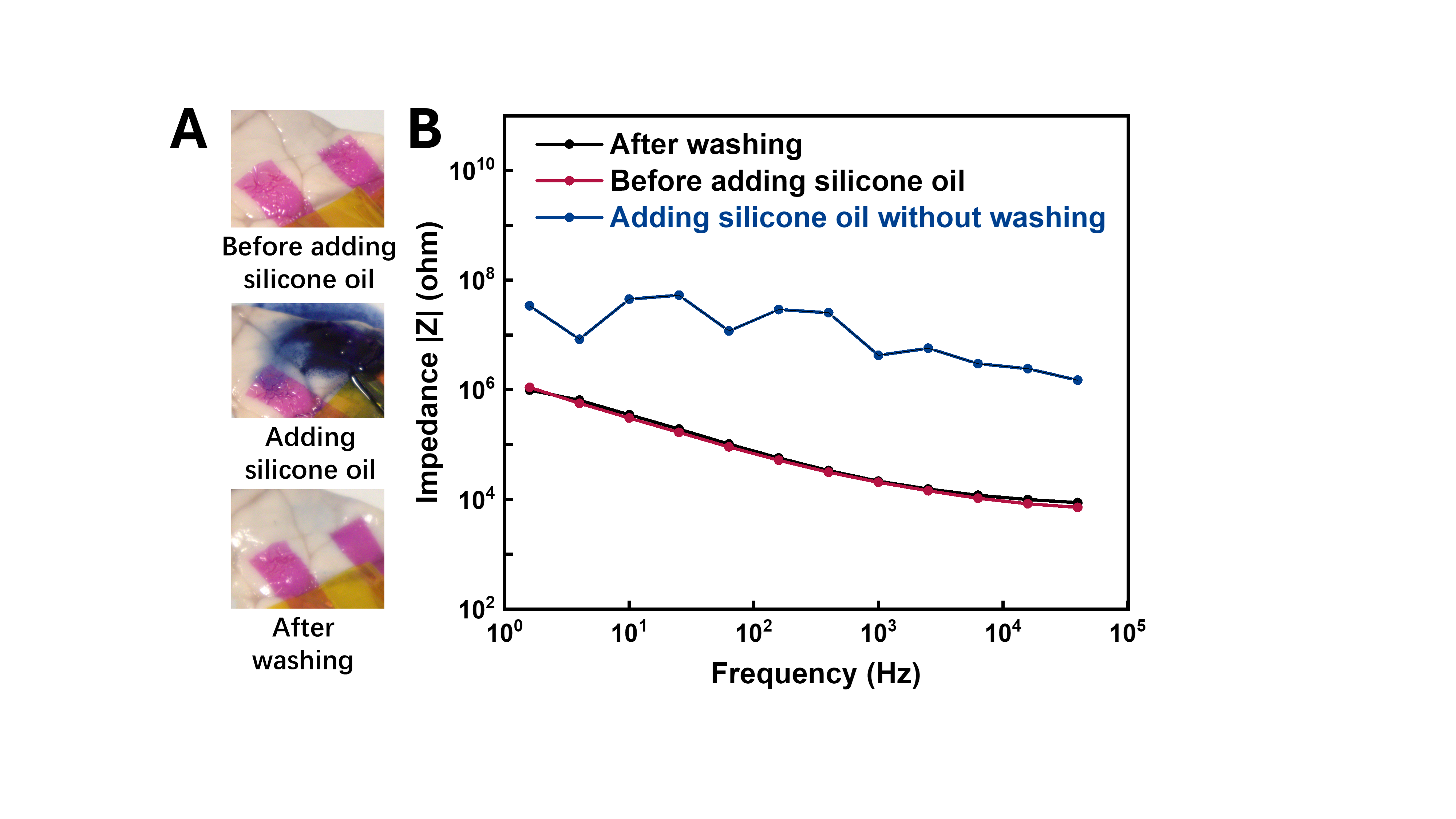


**Figure S16. Electrical impedance spectroscopy (EIS) measurements to characterize silicone oil removal efficiency.** (A) The photographs of tissue before adding silicone oil, adding silicone oil without washing and after washing. Blue dye was added to silicon oil for visualization (B) Electrical impedance spectroscopy (EIS) of STICH at the interface before adding silicone oil, adding silicone oil without washing and after washing.


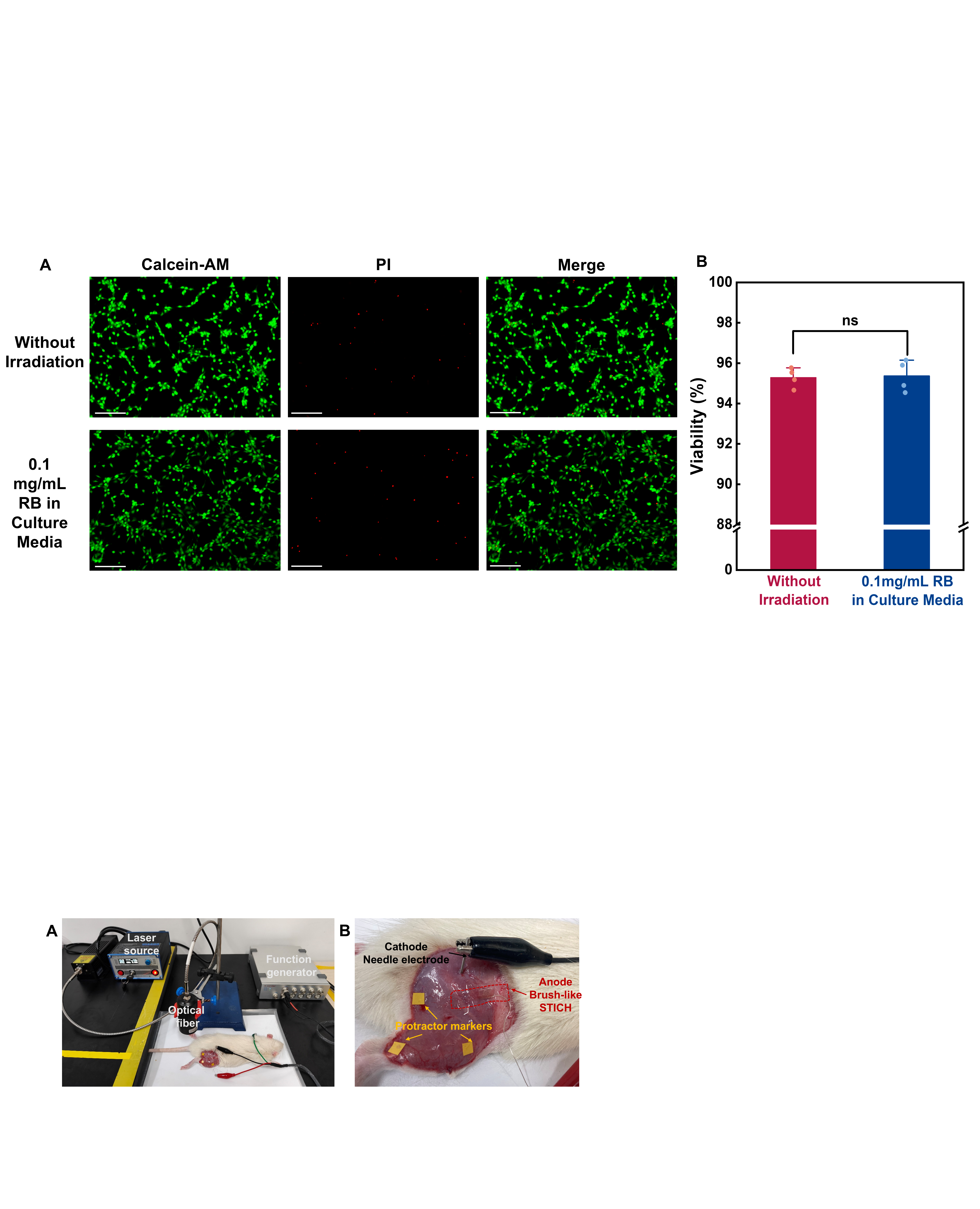


**Figure S17. In vitro biocompatibility assessment of STICH without irradiation and in Rose Bengal-containing environment for 24h.** (A) Live (green) and dead (red) fluorescence images of fibroblasts cultured for 24 h on STICH without irradiation and on well plates containing 0.1 mg/mL RB in the culture media. Scale bars: 200 μm. (B) Viability of cells cultured on STICH without irradiation and on well plates containing 0.1 mg/mL RB in the culture media (mean ± SD, n=4). Significance is evaluated by an unpaired two-samples *t*-test; ns, *p* > 0.05.


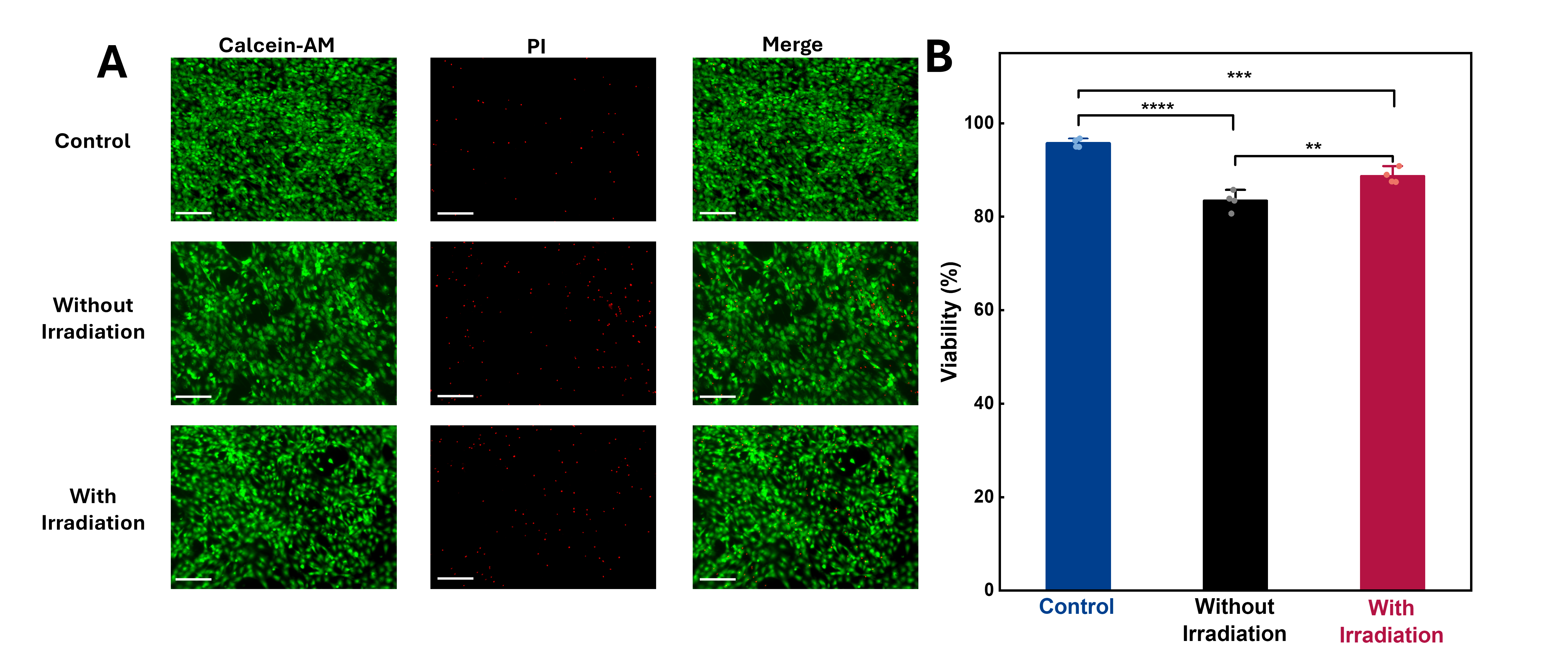


**Figure S18.** **In vitro biocompatibility assessment of STICH under different conditions for 7 days.** (A) Live (green) and dead (red) fluorescence images of fibroblasts cultured for 7 days on STICH without irradiation, with irradiation and on well plates. Scale bars: 200 μm. (B) Viability of cells cultured on STICH under three conditions (mean ± SD, n=4). Significance is evaluated by one-way ANOVA test; ***, p < 0.01; ***, p < 0.001; ****, p < 0.0001*. The 7-day viability of cells cultured on the hydrogel films remained well above 70%, supporting that the STICH interface is non-cytotoxic and biocompatible.


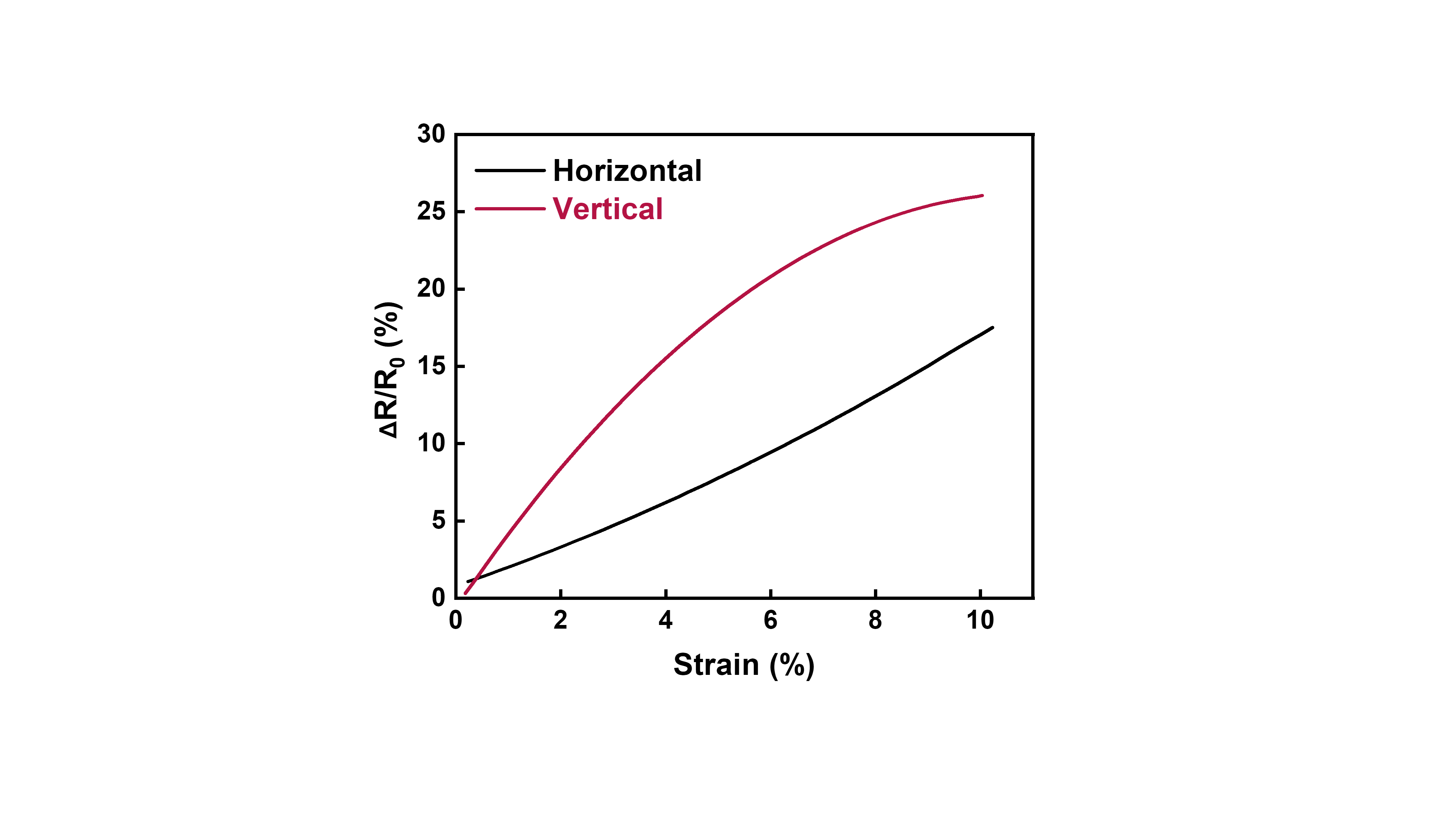


**Figure S19. Relative resistance change (ΔR/R₀) of the STICH device under horizontal and vertical tensile strain.**

**
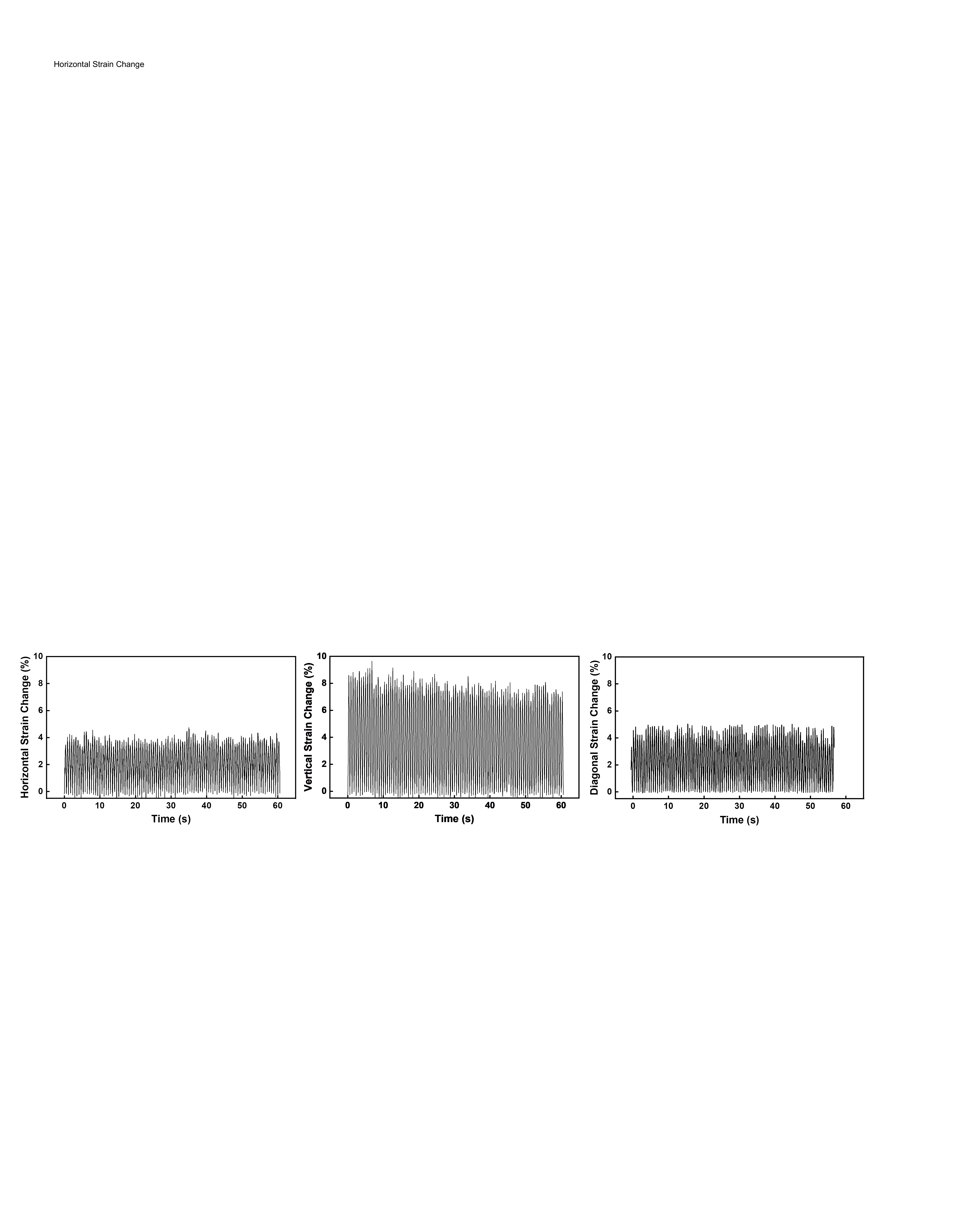
**

**Figure S20. Quantification of heart deformation along three anatomical directions based on video analysis.** Horizonal, vertical and diagonal strain variations along longitudinal, circumferential, and oblique directions of the heart were measured using video analysis software from recorded deformation during cyclic pumping.

**
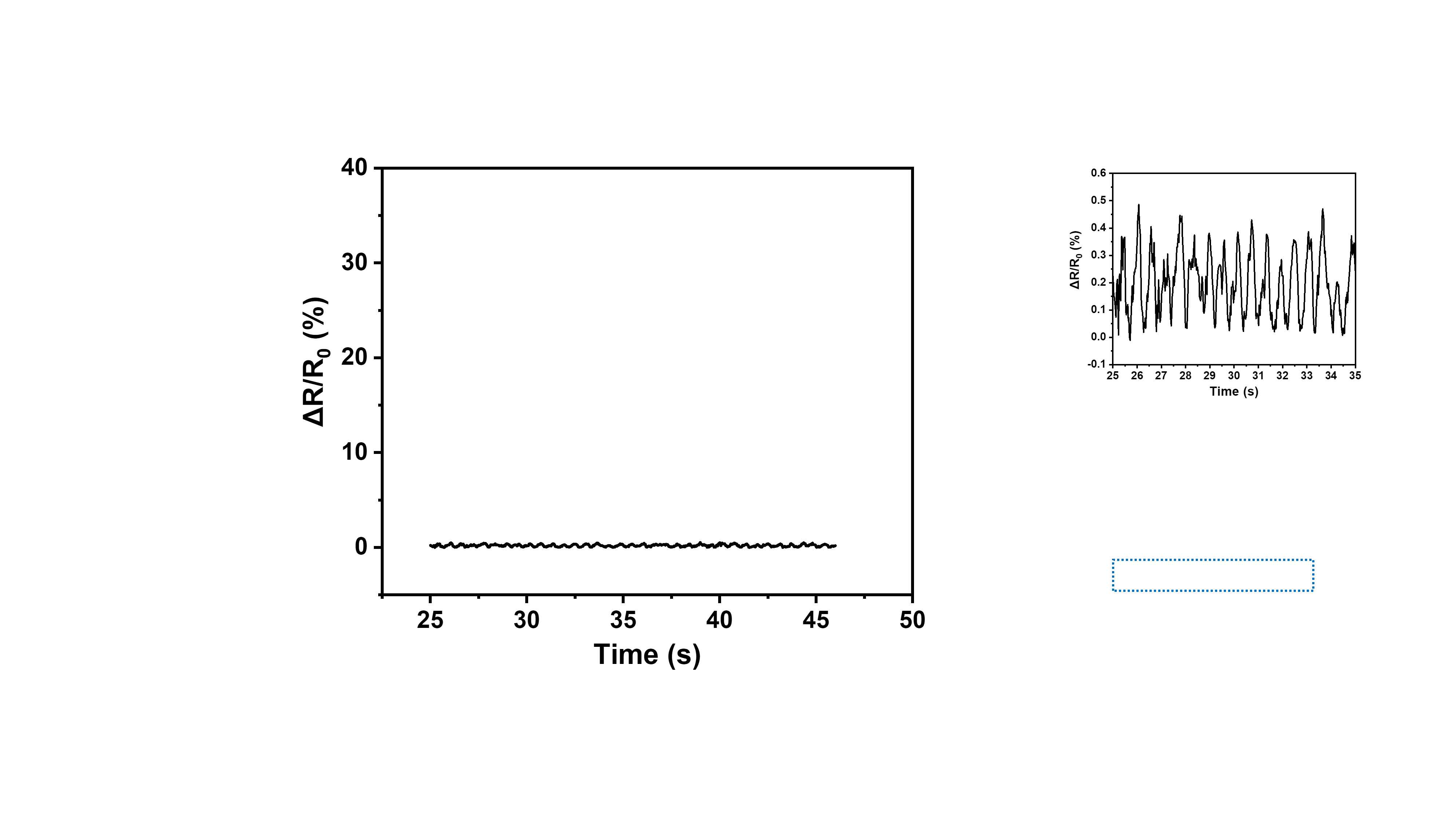
**

**Figure S21. Representative resistance changes-time curve of not spatially programmed STICH on *ex-vivo* porcine heart model.**


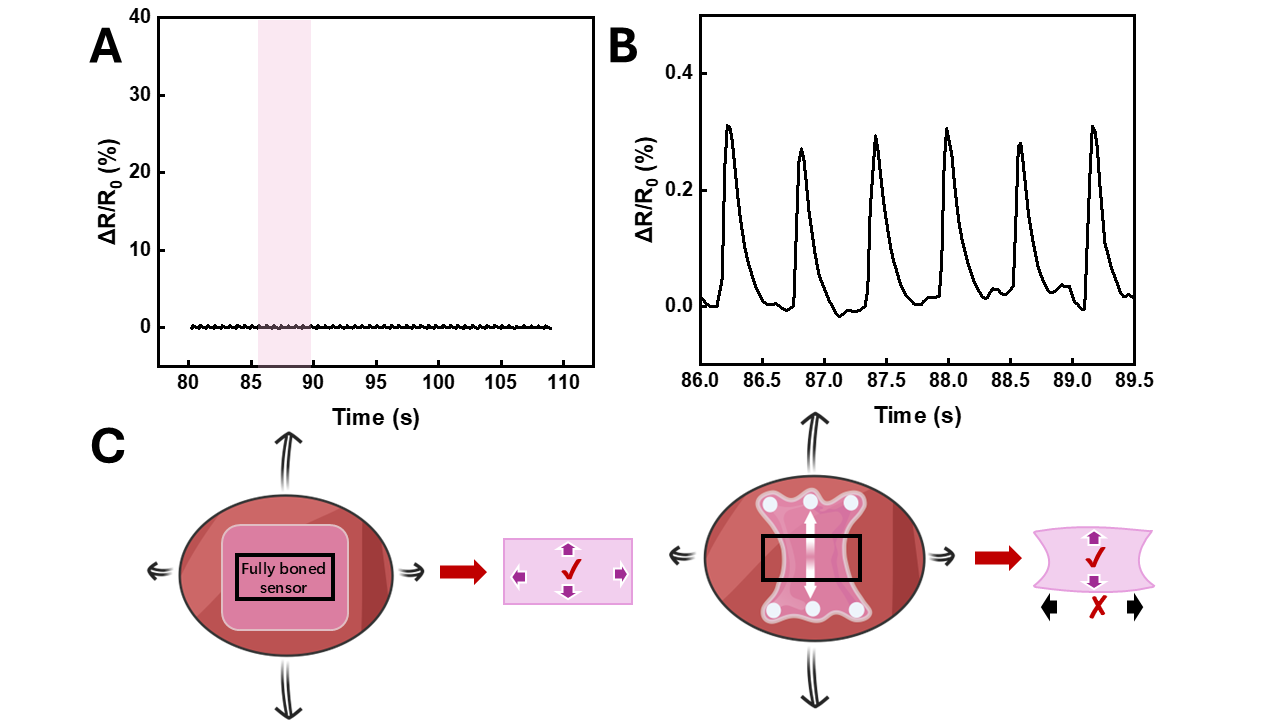


**Figure S22.** **Resistance changes of fully bonded STICH sensor on *ex-vivo* porcine heart model.** (A) Representative resistance changes over time for fully bonded sensor on *ex-vivo* porcine heart model. (B) The resistance changes during 5 cycles corresponding to the pink area in the (A). (C) A schematic illustration showing the fully bonded STICH sensor is sensitive to all directions, while the spatially patterned sensor is not sensitive to horizontal strain changes.

**
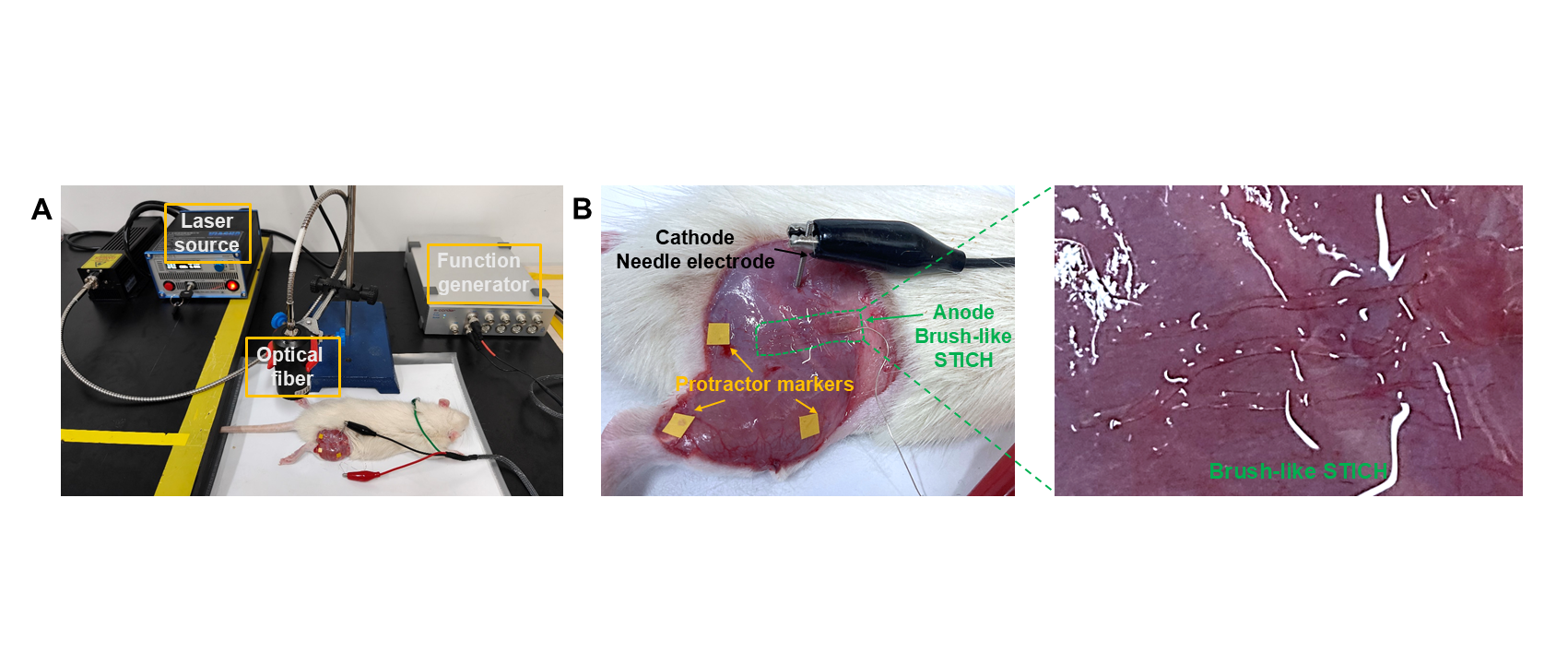
**

**Figure S23. *In vivo* experimental setup for muscle stimulation using the STICH device.** (A) System overview showing the green laser source connected to an optical fiber, and the monophasic function generator used for stimulation signal output. (B) Zoomed in view of surgical site with the brush-like STICH electrode adhered as the anode on the proximal thigh. Protractor markers beneath the leg aid in quantifying joint movement during stimulation.


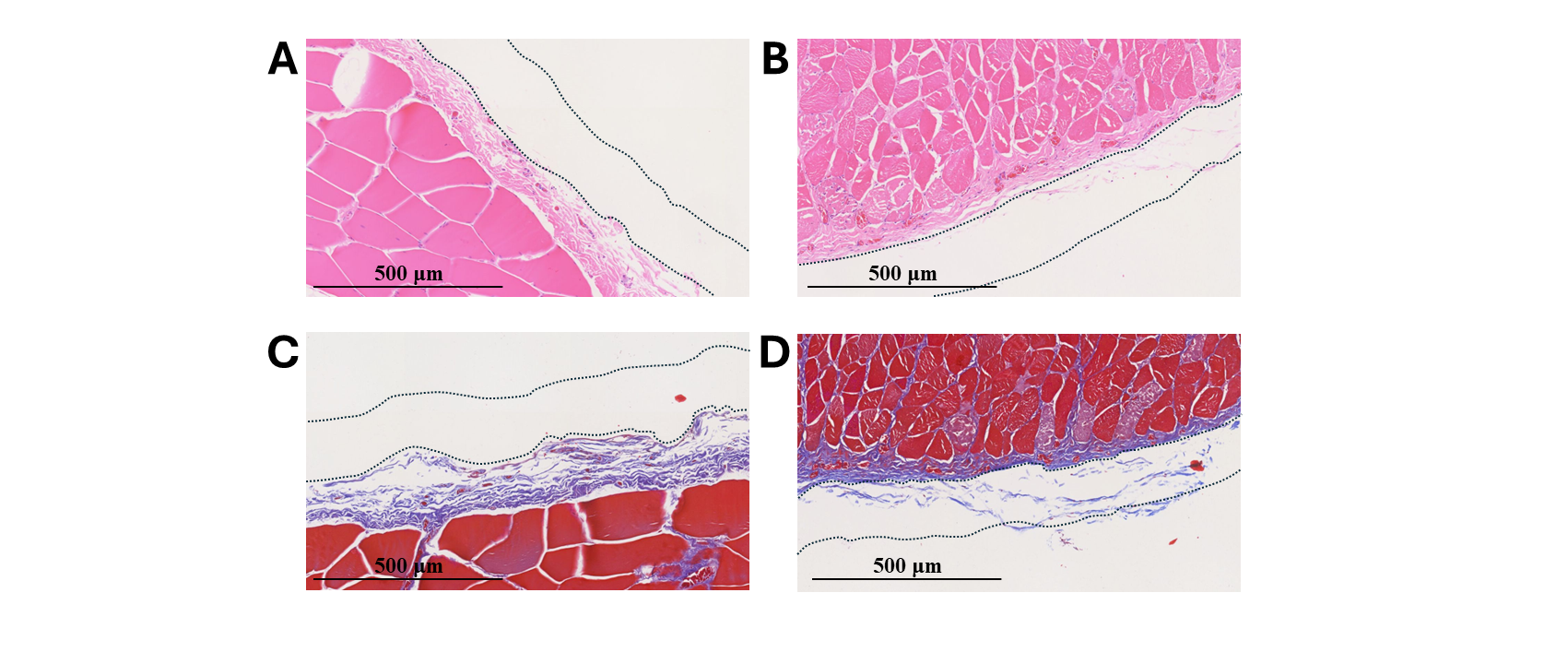


**Figure S24. H&E staining and Masson’s trichrome staining of adhesive interface.** (A) H&E staining of adhesive interface after irradiation at Day 0. (B) H&E staining of adhesive interface after irradiation for 7 days post-implantation. (C) Masson's trichrome staining of adhesive interface after irradiation at Day 0. (D) Masson's trichrome staining of adhesive interface after irradiation for 7 days post-implantation. (The dash lines show the position of STICH interface).

**References:**

[1] S. Li, C. Schöneich, R. T. Borchardt, *Biotechnology and Bioengineering* **1995**, *48*, 490.

[2] E. R. Stadtman, *Annual Review of Biochemistry* **1993**, *62*, 797.

[3] J. Uranga, J. I. Mujika, R. Grande-Aztatzi, J. M. Matxain, *J. Phys. Chem. B* **2018**, *122*, 4956.

[4] R. W. Redmond, I. E. Kochevar, *Photochemistry and Photobiology* **2019**, *95*, 1097.

[5] J. Yu, R. Wan, F. Tian, J. Cao, W. Wang, Q. Liu, H. Yang, J. Liu, X. Liu, T. Lin, J. Xu, B. Lu, *Small* **2024**, *20*, 2308778.

[6] C. Yu, M. Shi, S. He, M. Yao, H. Sun, Z. Yue, Y. Qiu, B. Liu, L. Liang, Z. Zhao, F. Yao, H. Zhang, J. Li, *Nat Commun* **2023**, *14*, 6226.

[7] J. Deng, H. Yuk, J. Wu, C. E. Varela, X. Chen, E. T. Roche, C. F. Guo, X. Zhao, *Nat. Mater.* **2021**, *20*, 229.

[8] S. J. Wu, J. Wu, S. J. Kaser, H. Roh, R. D. Shiferaw, H. Yuk, X. Zhao, *Nat Commun* **2024**, *15*, 1215.

[9] C. Yu, Z. Yue, M. Shi, L. Jiang, S. Chen, M. Yao, Q. Yu, X. Wu, H. Zhang, F. Yao, C. Wang, H. Sun, J. Li, *ACS Nano* **2022**, *16*, 16234.

[10] X. Wang, X. Sun, D. Gan, M. Soubrier, H.-Y. Chiang, L. Yan, Y. Li, J. Li, S. Yu, Y. Xia, K. Wang, Q. Qin, X. Jiang, L. Han, T. Pan, C. Xie, X. Lu, *Matter* **2022**, *5*, 1204.

[11] J. Chong, C. Sung, K. S. Nam, T. Kang, H. Kim, H. Lee, H. Park, S. Park, J. Kang, *Nat Commun* **2023**, *14*, 2206.

[12] A. Inoue, H. Yuk, B. Lu, X. Zhao, *Science Advances* **2020**, *6*, eaay5394.

[13] Y. Wang, P. Song, L. Wu, Z. Su, X. Gui, C. Gao, H. Zhao, Y. Wang, Z. Li, Y. Cen, B. Pan, Z. Zhang, C. Zhou, *J Mater Chem B* **2023**, *11*, 837.

**Captions for Supplementary Videos**

**Supplementary Video 1 |** Mechanoelectrical sensing with STICH on an *ex vivo* beating porcine heart model.

**Supplementary Video 2 |** Leg movement in response to electrical stimulation following three irradiation patterns.

**Supplementary Video 3|** STICH removal from adhered tissue using NaHCO_3_ treatment.
